# Roles of biomaterials in modulating the innate immune response in ocular therapy

**DOI:** 10.3389/fddev.2023.1077253

**Published:** 2023-02-15

**Authors:** Mehrnoosh Rafiei, Jin Teng Chung, Ying Chau

**Affiliations:** ^1^ Department of Chemical and Biological Engineering, The Hong Kong University of Science and Technology, Hong Kong, China; ^2^ Institute for Nanoscience and Nanotechnology, Sharif University of Technology, Tehran, Iran

**Keywords:** biomaterials, eye, immune-related disorders, innate immune system, immunomodulation

## Abstract

The eye is a hard-to-treat organ due to its poor regenerative capacity and susceptibility to inflammation; as a result, it has an immune privilege mechanism. In the case of ocular degenerative disorders, chronic and uncontrolled ocular inflammations can overcome this immune response to initiate and exacerbate tissue degeneration, ultimately leading to blindness. Recent landmark discoveries on the key roles of the ocular innate immune system in regulating acute and chronic inflammations as well as tissue fibrosis and homeostasis have shed light on the value of novel treatment interventions in modulating ocular immune responses at the molecular, cellular, and tissue levels. This strategy can be attained by using therapeutics to target resident phagocytes and antigen-presenting cells, namely, microglia and dendritic cells, as well as infiltrating neutrophils and macrophages. Biomaterials are foreign materials to the host and interact with innate immune cells. To leverage such intrinsic immunomodulatory properties, biomaterials such as implants, injectable depots, and nano/micro particles can be used alone as a treatment or with different payloads as carriers in immune-related ocular disorders. This article discusses how physicochemical properties such as biodegradability, size, shape, and charge affect biomaterials’ interaction with the eye’s innate immune system, therefore influencing outcomes towards pro- or anti-inflammatory responses. Knowledge about the eye’s immunological response is required for designing tolerogenic biomaterials including intraocular lenses, cellular scaffolds, therapeutic molecule depots, or carriers of gene therapies. The discussion presented in this review will shed light on the potential use of biomaterials to direct immune responses toward favorable treatment outcomes.

## 1 Introduction

Since eye tissue, which serves as our window to the outside world, lacks the ability to regenerate, immune-related inflammation poses a serious risk to the eye by potentially damaging its tissue, leading to vision loss. As a result, ocular tissue employs an immune privilege strategy to actively prevent any inflammation by providing an immunosuppressive environment (Niederkorn, 2019; Murakami et al., 2020). The term “immune privilege” comes from Medawar and others’ definition in the 1940s (Medawar, 1948; Taylor, 2016), where the ocular anti-inflammatory mechanism of having a private microenvironment for improving the anti-inflammatory response and tolerating the ocular immune cell’s function and balancing it was discussed. Eye tissue, in homeostatic conditions, has an immunoregulatory function (Dick et al., 2003). This inherent ocular immune tolerance, however, is compromised by several degenerative disorders including uveitis, diabetic retinopathy (DR), dry eye disease (DED), age-related macular degeneration (AMD), and choroidal neovascularization (CNV) (Perez and Caspi, 2015; Murakami et al., 2020; Gilger and Hirsch, 2022).

The body’s immune system response battles danger agents in two ways: the innate immune response and the adaptive immune response (Dempsey et al., 2003). In this way, the innate immune system acts as a first defender during the first days of the inflammation process or injury. If the innate immune cells are activated and do not regulate by proper signaling pathways after treatment, chronic inflammation and even visual impairment may occur (Murakami et al., 2020).

Different innate immune cells, including neutrophils, microglia/macrophages, and dendritic cells in the eye, play essential roles in the aforementioned types of diseases. The infiltration and activity of innate immune cells change depending on the type of ocular disorder, its location, and the milieu supplied by the cells (reviewed in detail by (Murakami et al. (2020)), in the form of inflammatory induction or suppression. When eyes are infected or injured, different proinflammatory cytokines and chemokines (such as tumor necrosis factor-alpha (TNF- α ), interleukin 8 (IL8), IL6, IL12, C-X-C motif chemokine ligand 10 (CXCL10), and C-C motif chemokine ligand 2 (CCL2), *etc.*) are produced by innate immune cells (Das et al., 2022). For uveitis, literature reports have shown that macrophages are essential innate immune cells in the development and resolution of experimental autoimmune uveitis. Macrophages can impact both proinflammatory side (by TNF- α activation) and immunosuppressive side [by RANTES (CCL5) production that is responsible for recruiting CD8^+^ T (killer T) cells] (Mérida et al., 2015; Yang et al., 2016; Murakami et al., 2020; Kubota et al., 2022). In a study on ocular infiltration of macrophages in experimental autoimmune uveitis (EAU), it was found that 1 week (day 16) after the beginning of macrophage infiltration in the eye (day 9), the inflammation reached a maximum amount (Sonoda et al., 2003). Monocytes from the peripheral blood infiltrate and differentiate into macrophages in degenerative retinal diseases with a reduced blood-retinal barrier function (Ginhoux et al., 2013). In the aging retina and choroid, inflammatory macrophages from monocytes, dendritic cells, and tissue-resident macrophages such as microglia are important immune cells. These immune cells are involved in the pathogenesis of AMD, and researchers observed infiltration of macrophages and lymphocytes around the CNV area, as well as an elevated amount of CCL2, IL-8, and vascular endothelial growth factor (VEGF) in circulation blood monocytes (Lopez et al., 1991; Lechner et al., 2017; Chen et al., 2019; Das et al., 2022). A recent review by Das et al. provides more information on the effects of aging on the function of immune cells during ocular disorders (Das et al., 2022). In DR, activated neutrophils and macrophages can cause retinal vascular injury. Moreover, the number of immune cells in the choroid of DR patients was elevated (Schroder et al., 1991; Lutty et al., 1997).

**FIGURE 1:**
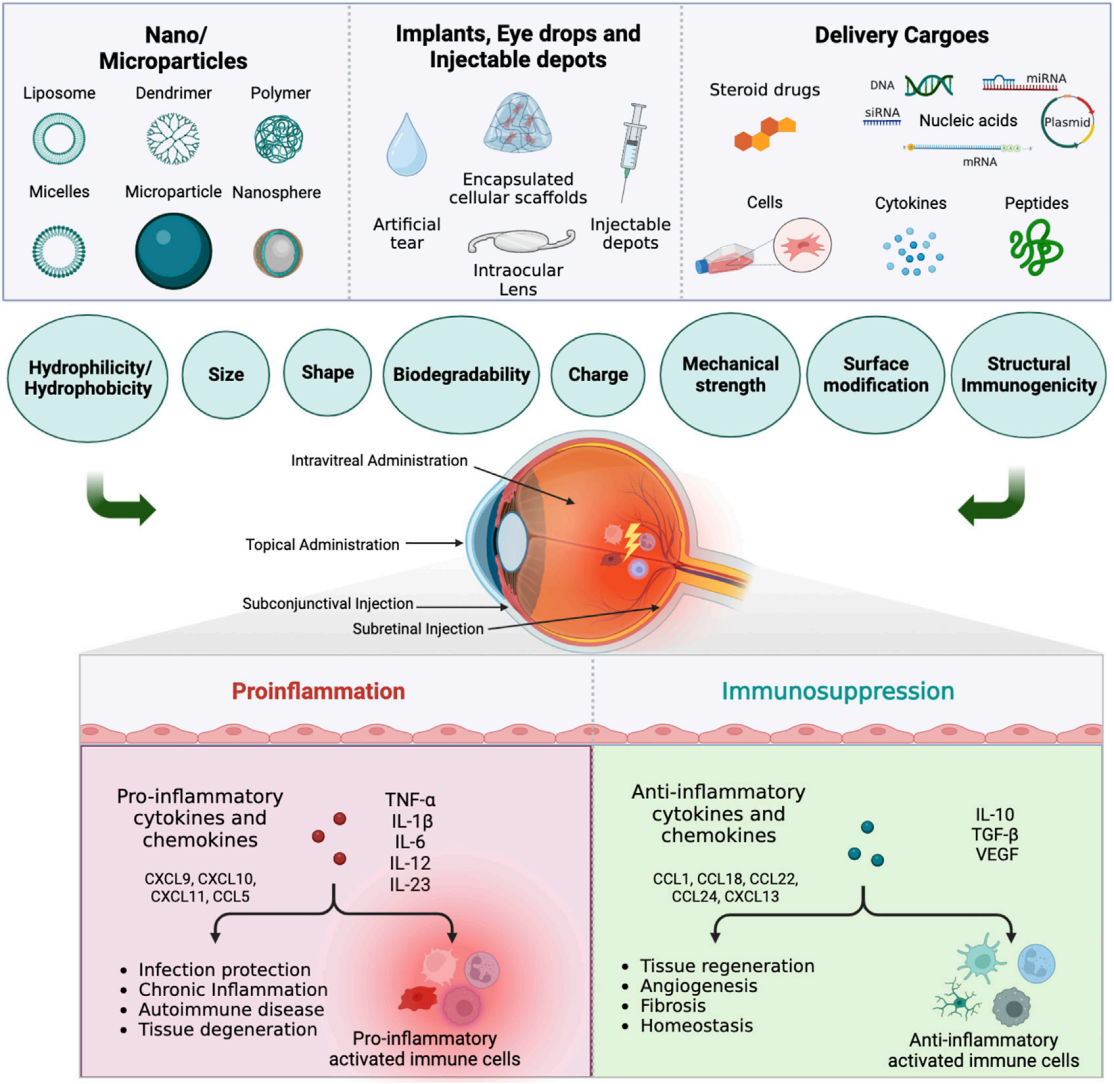
Immunomodulatory drug delivery systems for ocular innate immune cells immunotherapy. Biomaterials such as nanoparticles, microparticles, implants, eye drops, and injectable depots, with or without immunomodulatory cargoes, can be used to treat ocular diseases *via* different administration routes. Depending on the physiochemical properties, the biomaterials can promote or suppress the response from proinflammatory innate immune cells, which will in turn affect the therapeutic outcome (Created in BioRender.com).

The biocompatibility of a material is defined by William’s dictionary as “the ability of a material to perform with an appropriate host response in a specific application” (Donaruma, 1988; Williams, 1999). Biomaterials can act alone as a treatment or can be combined with other modalities to provide therapeutic intervention. The roles of biomaterials in immunomodulation in ocular therapy can be classified as follows. The first approach is to use biomaterials to construct carriers of therapeutic agents while the biomaterial itself is intended to passively and inertly affect the immune response (Garzón et al., 2022). The second approach is similar but more intriguing. Also for constructing carriers, the biomaterial is designed to interact with the immune system, to promote the immunogenic or immunotolerant effect, depending on the application, and to augment the effect of the cargo (Im, 2020; Gao et al., 2022). For these two approaches, the immunomodulatory cargoes include steroids, proteins, and nucleic acids. In a third approach, biomaterials are used to make ophthalmic devices such as ocular lenses and ocular inserts (Kwon et al., 2020). The functions and longevities of these devices in part depend on their interaction with the immune system. The last approach is to use biomaterials to support cell therapy. There are several reviews on the design criteria of immunomodulatory biomaterials in tissue engineering (Zolnik et al., 2010; Andorko and Jewell, 2017; Li H. et al., 2022; Mitarotonda et al., 2022). One possible therapeutic intervention is to direct the response of immune cells by using immunomodulatory biomaterials alone or in combination with engineered cells. An overview of these approaches is summarized in Figure 1. The biomaterials can be designed to participate in the immunogenic or immunosuppressive pathways based on their physicochemical properties such as form, size, shape, charge, hydrophilicity/hydrophobicity, degradability, and mechanical strength. In this review, we will focus on the interaction of biomaterials with the eye’s innate immune system, and consider how they can influence, positively or negatively, the treatment of inflammatory ocular diseases.

## 2 The innate immune system in the eye

**FIGURE 2:**
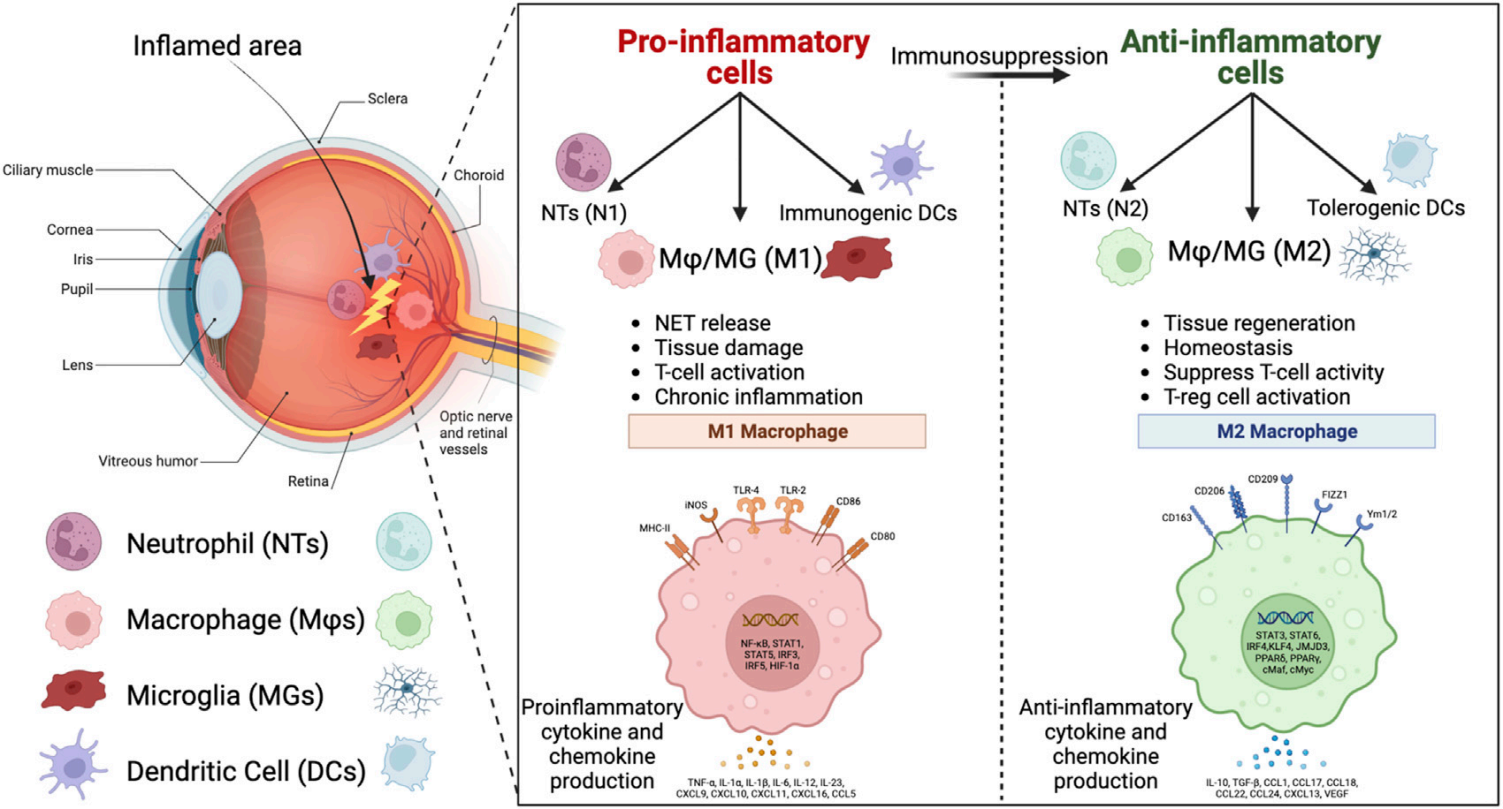
Eye tissue and the key innate immune cells in the inflamed area. The pro-inflammatory innate immune cells, including N1 neutrophils (NTs), M1 macrophages/microglia (MΦ/MG), and immunogenic dendritic cells (DCs), with proinflammatory functions that are listed in the left window, can be re-educated by immunomodulatory biomaterials to anti-inflammatory cells, including N2 NTs, M2 MΦ/MG, and tolerogenic DCs, with anti-inflammatory functions that are listed in the right window (Created in BioRender.com).

Eye tissue has been recognized as an immune-privileged organ for 150 years. The first long-term survival of mouse skin implanted into a dog eye’s anterior chamber was described by a Dutch ophthalmologist (van Dooremaal, 1873). Zirm carried out the first successful human corneal transplant a few years later, in 1905 (Zirm, 1906; Niederkorn, 2019). At the time of these observations, neither the human immune system nor the fundamentals of implant rejection had been studied. By inserting rabbit skin into an allogenic rabbit’s anterior chamber, Medawar further demonstrated in 1948 the distinct immunologic characteristics of the eye (Medawar, 1948). Immune privilege is an action in the homeostatic condition of the eye that modulates the induction and progression of inflammation to prevent any excessive inflammation that could degenerate tissue (Murakami et al., 2020). Ocular immune privilege consists of an immunosuppressive microenvironment with different components including transforming growth factor-β (TGF- β ) (Taylor and Ng, 2018), retinoic acid (Zhou et al., 2011), programmed cell death-ligand 1 (PD-L1) (Sugita et al., 2009), galectins (Toscano et al., 2006), and cluster of differentiation-200 receptor (CD200R), *etc.* (Dick et al., 2003; Murakami et al., 2020). The innate immune cells are dependent on these ocular microenvironment signals to destroy or regenerate tissue during inflammation (Murakami et al., 2020). After infection, injury, or even using implants in the eye, neutrophils, macrophages/microglia cells, and dendritic cells attack the site of inflammation and start secreting proinflammatory cytokine and chemokines. In this way, proinflammatory-activated macrophages, broadly classified as “M1 macrophages”, secrete proinflammatory cytokines including IL1-β, IL12, inducible nitric oxide synthase (iNOS), and TNF-α. The M1 macrophages kill the pathogens or prepare a fibrotic capsule around the foreign implant, recruit leukocytes to the site, and activate the adaptive immune system. This process spans from the first days to weeks, depending on the damage and implanted biomaterial. Afterward, the macrophage cells’ phenotypes change to the characteristics of “M2 macrophages”, which regenerate and heal the area by angiogenesis and by secreting anti-inflammatory cytokines such as IL10 and TGF- β (Anderson, 2003; Mosser and Edwards, 2008; Andorko and Jewell, 2017). As a result, the phenotypic diversity of macrophages with distinct markers and functions can be targeted using biomaterials with specific physiochemical properties to achieve the desired inflammatory function. The M1 markers can be used to upregulate the inflammatory functions and the M2 markers can be used to downregulate the inflammatory function of macrophages (Figure 2). In the same way as macrophages, neutrophils respond to extracellular stimuli in a context-dependent manner and can polarize from N1 to N2 phenotypes, similar to the M1 and M2 phases of macrophages (Cuartero et al., 2013; García-Culebras et al., 2018). In this review, our focus is on downregulation of the innate immune system inflammatory response in the case of ocular inflammation by using modulatory biomaterials in different forms such as implants, injectable depots, nano/micro systems, and hybrids of these systems (Figure 1).

### 2.1 Key players as innate immune cells in the eye

#### 2.1.1 Neutrophils

The main players in acute inflammation and the first line of fighting any danger, including pathogens, inflammation, and injury in the innate immune system, are neutrophils. Neutrophils’ lifespan has been shown to be very short (less than 24 h) in mice and humans, although recently, a number of studies showed that the lifespan of neutrophils can be increased by inflammatory cytokines and signals that induce the adaptive immune system and aggravate the inflammation for up to 5.4 days (Pillay et al., 2010; Kolaczkowska and Kubes, 2013). They are the first immune cells that detect any inflammation that occurs, by sensing the pathogen-associated molecular pattern (PAMP) present in microbes and the danger-associated molecular pattern (DAMP) present in injured tissues. Neutrophils play a critical role in the pro-inflammatory function through phagocytosis and by secreting pro-inflammatory cytokines to induce an innate and adaptive immune response (Ghosh et al., 2019). Neutrophils also release neutrophil extracellular traps (NETs). NETs are composed of nuclear DNA, histones, and other granular proteins. NETs are induced by nitric oxide, cytokines, and other autoantibodies to prevent inflammation progression. However, it has been shown that chronic aggregation of NETs can cause autoimmune, allergic, chronic diseases, tissue damage, and immune rejection to implants (Kolaczkowska and Kubes, 2013; Mahajan et al., 2021; Mun et al., 2021; Yıldız and Yıldız, 2022). For example, Mahajan et al. (2021) found that NETs aggregated after ocular surface inflammation, resulting in meibomian gland dysfunction (MGD) due to blockage. Although it has been shown that NETs are useful to prevent inflammation progression in the eye rheum, they are harmful to corneal diseases, uveitis, and diabetic retinopathy and contribute to poor prognosis of these diseases (Estúa-Acosta et al., 2019). Neutrophils perform diversely in inflammation and injury conditions by regulating acute inflammation and repair processes, autoimmune diseases, and chronic inflammatory diseases, based on the type of cytokines and special receptors that exist in the inflamed environment (Estúa-Acosta et al., 2019; Liew and Kubes, 2019; Tan et al., 2020). The signaling pathways of an inflamed environment can result in different phenotypes of neutrophils: N1, which represents the proinflammatory phase, and N2, which represents the anti-inflammatory phase (Cuartero et al., 2013; García-Culebras et al., 2018).

#### 2.1.2 Microglia/macrophages

Macrophages are key players in the innate immune system, fighting any infection and danger in the tissue through phagocytosis and by inducing regeneration and fibrosis afterward. They function in a spectrum between the M1 proinflammatory phase and the M2 anti-inflammatory phase (Tan et al., 2020). The interferon‐gamma (IFN- γ ), TNF- α , IL-1 β , IL12, IL6, and lipopolysaccharides (LPS) signals can activate the M1 macrophage phase and iNOS M1 activated cell expresses surface markers, cluster of Differentiation 36 (CD36), CD86, CD80, C-C chemokine receptor type 7 (CCR7), and major histocompatibility complex class II (MHCII) (Figure 2). On the other hand, the M2 macrophage phase is activated by signaling factors such as IL-4, IL13, TGF- β , and IL10, and the M2 activated cell expresses surface markers of CD206, CCL204, and CD163. Throughout the eye, they can be found in the anterior and posterior parts (cornea, iris, ciliary body, choroid, sclera, and vitreous), and play a critical role in controlling inflammation and injury in the first days (Chinnery et al., 1947; Ghasemi et al., 2012). For instance, vitreal macrophages (also known as hyalocytes) are located between the inner limiting membrane of the retina and the vitreous membrane that contains condensed vitreal collagen (Chinnery et al., 1947).

Retina tissue in the eye possesses resident immune cells known as glial cells, which are categorized into three main groups: astrocytes, microglia, and Müller glia. Microglia cells are myeloid-derived populations in the retina that manage the immune response and environmental cues by interacting with retinal cells and maintaining the retinal cell’s homeostatic function and neuronal homeostasis (Tan et al., 2020; Guo et al., 2022; Wang and Cepko, 2022). They play a critical role in interacting with retinal cells and recruiting neutrophils and macrophages for further functions during eye injuries and inflammation (Minghetti et al., 2008; Tan et al., 2020). The resting state of microglia cells is achieved by intraretinal signaling and cytokines secretion such as TGF- β and IL-10 and this state has a low expression of membrane receptors (Langmann, 2007). They are sensitive and can be activated through toll-like receptor (TLR) signaling with a variety of triggers, including LPS, calcium level fluctuations, proinflammatory cytokines (IFN- γ , TNF- α , *etc.*), thrombin, aggregates, peptides derived from ocular infections, autoimmune processes, ischemia, neuronal damage, and neurodegeneration-associated signals. Microglia cells are the first players in reacting to injury or inflammation at retina or ocular sites (Langmann, 2007; Minghetti et al., 2008). In the first 24 h of microglia activation, cell proliferation, morphology transition to ameboid shape, migration, phagocytosis, upregulation of immunoglobulin G (IgG), CD1 receptors, and secretion of different proinflammatory signals such as cytokines (IL1- β , IL6, TNF- α ), chemokines (CCL3 (MIP-1 α ), CCL4 (MIP-1 β ), CCL2), and other signals (NO, ROS) occurs (Fan et al., 2022). Neutrophils and macrophages are recruited according to the proinflammatory signal’s level. The P2 purinergic receptor (P2Y12) is a phagocytic receptor expressed on microglia that responds to tissue damage by sensing ADP/ATP (Adenosine Di/Tri-Phosphate). This receptor has been shown to be necessary for the clearance of apoptotic cells, and inhibiting it increases the clearance time (Blume et al., 2020). A microglia is characterized by three distinct phases: the resting phase with ramified shapes, the activated pro-inflammatory phase or M1 phase with ameboid, and the anti-inflammatory phase (M2) with ramified shapes. Activation of microglia to the M1 phase occurs when infection, inflammation, or a local ocular injury takes place in the eye tissue. The activated microglia rapidly migrate to the site of inflammation or injury and secrete proinflammatory signals including TNF-α, cyclooxygenase-2 (COX-2), and iNOS to recruit peripheral immune cells (monocytes). Afterward, microglia and recruited macrophages fight the foreign harmful agent using hypo phagocytic functions. Following microglia and macrophage phagocytosis and removal of debris *via* M1 activation, the M1 activation phase transitions to the M2 anti-inflammatory phase by passing the phenotype from M1 to M2. Arginase 1 (ARG 1), CD206, and IL-10 are among the regenerating and angiogenesis factors that this M2 phenotype secretes, helping to return the inflamed area to homeostasis (Tan et al., 2020). However, chronic activation of microglia is one of the major players in the development of neurodegenerative diseases, including glaucoma, wet AMD, and DR, by the barrier breakdown mechanism (Reichenbach and Bringmann, 2020). The markers for the different phases of microglia are identical to those for monocyte-derived macrophages (TNF- α , COX-2, and iNOS), in addition to specific surface markers for differentiating from monocyte-derived macrophages, such as P2Y12 for the resting and M2 phases of microglia and ionized calcium-binding adapter molecule 1 (Iba-1) protein marker for the phagocytotic and activated microglia (Ito et al., 1998). Some reports claim that the expression of the Iba-1 surface marker is associated with the microglia’s phagocytosis phase (Ohsawa et al., 2000; Morillas et al., 2021). Between microglia and retinal cells, a variety of ligand-receptors exist that inhibit microglia activation and restore their resting phase and homeostasis state. For instance, TGF- β signaling is used to downregulate the MHC-II, CD80, and CD86 receptors, which are expressed on the surface of M1 microglia; glycoprotein CD200 (known as mAb OX2) is detected by CD200R (a myeloid-specific receptor on microglia); CD200-CD200R complex inhibits microglia activation; and chemokine fractalkine (CX3CL1) and CX3CR1 receptors produce a complex that inhibits the phagocytotic phase of microglia (Langmann, 2007; Minghetti et al., 2008).

Microglia activation is linked to several inflammatory and degenerative ocular disorders, including retinitis pigmentosa (RP), AMD, glaucoma, DR, and uveitis in the eye. This is due to their status as resident immune cells in the retina, which makes them very sensitive to changes in cell phenotype, ligand-receptor interactions, and environmental signals. For instance, it has been shown that in experimental AMD, aging CX3CR1 deficiency causes microglia to migrate and accumulate in the subretinal space because it lacks the CX3CR-1-CX3CL1 complex, which physiologically maintains microglia homeostasis (Penfold et al., 2001; Minghetti et al., 2008). Arroba et al. studied the polarization dynamics of microglia in DR in *in vitro* and *in vivo* in mice and found that the microglia are initially in the M2 phase, but at more advanced stages of DR development, their phase shifts to M1 and chronic pro-inflammatory (Arroba et al., 1862). Since the progression of RP, AMD, glaucoma, DR, and uveitis has been related to the activation of microglia, these cell types are of great interest as a target for treating these diseases. There are two effective ways to accomplish this goal: either preventing their pro-inflammatory response or reprogramming them *via* gene therapy to downregulate the inflammatory response and immune cell recruitment (Arroba et al., 1862; Minghetti et al., 2008; Guo et al., 2022; Wang and Cepko, 2022).

The dynamics of microglia activation and repolarization to homeostasis, with Iba-1 (for M1 phase) and P2Y12 (for M2 phase) as markers, was monitored in an ocular hypertension (OHT)-induced glaucoma mouse model by Ramírez et al. (2020). The authors showed that at first (day 0), the damaged tissue released ATP signals and P2Y12 was upregulated. After a few hours, the P2Y12 expression was downregulated, and this decreasing signal was the most sensitive alarm for the transition from the resting to the M1 phase of microglia with Iba-1+. After 24 h, the Iba-1+ cells showed P2Y12 expression elevation. On the 3rd and 5th days, downregulation of P2Y12 expression was observed. Based on their collected data, the authors stated that the inflammation peaked and was strongest on the 3rd and 5th days after induction. Then, P2Y12 expression increased slowly from day 8 to reach the naïve eye amount on the 15th day for this animal model. The same group conducted a different time point investigation on the levels of proinflammatory cytokines (IFN- γ , TNF- α , IL-1 β , IL6, IL12, IL17, and IL18) and anti-inflammatory cytokines (IL4, IL10, IL13, and TGF- β ) produced following OHT induction in the glaucoma, and found that IL6 expression peaked after 1, 3, and 5 days of induction. Interestingly, the authors discovered that IL4 and IL10 are important modulatory cytokines that allow activated microglia to repolarize to the M2 phase before further inflammation progression and tissue damage (Fernández-Albarral et al., 2021).

#### 2.1.3 Dendritic cells

Dendritic cells (DCs), similar to macrophages, are phagocytic and are able to present antigens to immune effector cells. The distinctive feature of DCs is their migration to lymph nodes to activate naïve lymphocytes, thereby bridging innate and adaptive immunity. Most DCs are found in the corneal and conjunctival epithelium, thereby serving as the first line of defense on the ocular surface. In the posterior ocular section, DCs can be mostly found in the retina and choroidal space (McMenamin et al., 2019). Once activated, most ocular DCs migrate to cervical lymph nodes to trigger downstream immune responses. There are several subtypes of DCs that can be found in the eye, such as conventional DCs (cDCs), Langerhans cells (LCs), plasmacytoid DCs (pDCs); they all play key roles in maintaining immune tolerance and resolving post-inflammation challenges.

In mice, cDCs can be classified into types 1 and 2. cDC1 can be identified as CD4^−^CD8α+CD11b-CD11c+ and cDC2 as CD4^+^CD8α-CD11b+CD11c+. cDCs recognize both extracellular and intracellular pathogens; of which cDC1 can efficiently cross-present exogenous antigens on MHC-I molecules to CD8^+^ T cells, whereas cDC2 activates CD4^+^ T cells, and trigger T_H_2 and T_H_17 immune responses. On the other hand, pDCs are distinguished from cDCs by the expression of CD45RA, lymphocyte antigen 6 complex (Ly6C), sialic acid binding immunoglobulin-like lectin H (Siglec-H), and CD317. These cells are important in anti-viral immunity and systemic autoimmunity, because they can sense intracellular self and non-self nucleic acids *via* TLRs pathways and produce type I and III interferons (Musumeci et al., 2019). In addition, LCs are another subtype of DC, which are considered to be potent APC. Under physiological conditions, their population frequency is 5%. However, during a disease/inflammation-challenged state, their population is replenished by infiltrating monocytes and 10%–20% of LCs migrate to the lymph nodes (Merad et al., 2008). LCs play a crucial role in maintaining immune resolution post-infection and tolerance. In the disease context of allergic contact dermatitis, it was observed that LCs can effectively induce anergy and apoptosis of CD8^+^ T cells while activating ICOS+ CD4^+^ FoxP3+ Treg cells (Kaplan et al., 2005).

Resident DCs are considered “immature” during the resting state. During the pathogen infection/inflammation state, DCs undergo maturation, expressing higher level of MHC-molecules, the co-stimulatory molecules CD86, CD83, CD40, and producing pro-inflammatory cytokines such as IL-12, IL-6, and TNF-α. Indeed, the activation of CD86^+^ DC participated in the onset and progression of dry eye disease (Maruoka et al., 2018). In addition, DCs were also found to promote the progression of anterior uveitis (Lin et al., 2019).

Under physiological conditions, ocular cells can actively participate in the suppression of DC immunogenic activation to maintain an anti-inflammatory state in the ocular environment. For instance, retinal pigment epithelial cells can produce IL-1Ra to suppress DC activation. Corneal stromal cells can produce TGF-β1 to inhibit DC activity while promoting corneal wound healing (Hamrah et al., 2003; Lu et al., 2012; Sugita et al., 2013; Morante-Palacios et al., 2021). In fact, DCs can also be activated into tolerogenic phenotypes. These DCs can produce anti-inflammatory cytokines such as IL-10, TGF-β, and indoleamine 2, 3-dioxygenase (IDO) to suppress T-cell activity and function in maintaining ocular tolerance. Tolerogenic DCs can be generated *in vitro* with the co-culture of stromal cells or by treatment with immunosuppressive agents such as IL-10, TGF-β, vitamin D receptor agonists, and vasoactive intestinal peptide (VIP) (Dempsey et al., 2003; Hu and Wan, 2011; Haneklaus et al., 2012; Perez and Caspi, 2015; Puri et al., 2022). Although tolerogenic DCs demonstrated therapeutic strengths in autoimmune diseases, the exploration of their potential in the ocular field is still under investigation.

## 3 Application of biomaterials in immunomodulation of the innate immune system

Biomaterials are widely used as carriers or scaffolds for ocular therapeutic entities, such as small molecular drugs, biomacromolecules such as proteins or nucleic acids and living cells. The carriers however have been known to actively interact with the immune system. The resulting host reaction as a result of the implanted materials is coined “foreign body response” (FBR). Biomaterial-induced FBR can lead to cargo clearance and elimination, countering therapeutic intentions. However, if properly gauged by appropriate material selection and design, the immune reaction can be utilized for pro-healing responses. Therefore, the design of biomaterials generally adopts strategies to 1) mitigate or 2) leverage FBR depending on the therapeutic goals in the eye. Indeed, this can be achieved with the high responsivity and plasticity of the above-mentioned innate immune cells in response to the exogenous biomaterials’ cues. With the appropriate selection of biomaterials and platform characteristics, these cells can be strategically manipulated and programmed toward a pro- or anti-immunomodulatory response to facilitate the overall therapeutic outcome.

Understanding the dynamics of FBR, that is, the biomaterials-innate immune cell interplay, is critical for this goal. The activation mechanism of innate immune cells by biomaterials is mainly governed by pattern-recognition receptors (PRRs). PRRs are present on the plasma membrane surface or in the cytoplasm and can sense a broad range of damage and pathogenic cues. PRRs consist of TLRs, nod-like receptors (NLRs), and inflammasomes. TLRs exist on the surface or in the endosomal compartment of antigen-presenting cells (APCs) including microglia, macrophages, and dendritic cells, and recognize a broad range of microbial molecules such as proteins, nucleic acids, and LPS, *etc.* NLRs are intracellular receptors with inflammasome subunits. Inflammasomes are the complex of these proteins, act as receptors of the innate immune system, and are responsible for the inflammatory response by cysteine-aspartic acid protease-1 (caspase-1) induction to produce the proinflammatory cytokines IL-1 β and IL-18. NLR1, NLR3, NLR4, and absent in melanoma 2 (AIM2) are different inflammasomes that play a key role in ocular inflammatory disorders such as glaucoma, DED, AMD, and DR in recent years (Yerramothu et al., 2018). It has been demonstrated in studies that dopamine, DMSO, microRNA-223 (miR223), and other inflammasome suppressors can lower the severity of inflammatory ocular illnesses. However, the mechanism by which inflammasomes function in the human body is unknown, necessitating more study to identify novel therapeutic approaches that focus on the inflammasome cascade (Bauernfeind et al., 2012; Haneklaus et al., 2012; Ahn et al., 2014; Yerramothu et al., 2018).

The activation of PRRs begins with the detection of foreign patterns including DAMP and PAMP and the secretion of signaling pathways to combat harmful agents (Andorko and Jewell, 2017). PAMPs and DAMPs can take in diverse forms; for instance, polysaccharides, peptides, glycopeptides, lipopeptides, LPSs, and nucleic acids, *etc.*, which are derived from microbes or damaged host cells fragments during invasion and tissue injuries. The eye’s innate immune system is extremely sensitive in detecting these molecular entities. Once the cells are activated, they secrete proinflammatory cytokines to initiate FBR. Similarly, the surfaces of biomaterials and delivery platforms also exhibit physicochemical features that resemble PAMPs. Therefore, FBR is inevitable with the introduction of ocular implants, injectable depots, and nano/micro particles. On the other hand, several TLRs are expressed by different ocular tissues, such as the cornea, conjunctiva, retinal pigment epithelial cells, and the uvea, to protect and isolate the eyeball from any specific PAMP (Wakefield et al., 2010). The uvea, for instance, is particularly susceptible to LPS-mediated TLR4, and acute anterior uveitis has been observed, based on TLR4 cell response to LPS (Yu and Hazlett, 2006). The ocular innate immune system, however, is also perceptive to anything that poses a threat to the host tissue through endogenous DAMPs. These molecules are released from the injured tissue or dead cells’ intracellular or extracellular regions including ATP, high mobility group protein B1 (HMGB1), and other molecules derived from injured tissue (Mahaling et al., 2022). According to the review by Mahaling et al. (2022)), these DAMPs are linked to inflammations brought on by age, increased ocular pressure, oxidative stress, ischemia, stress, and environmental factors, *etc.*, in retinal illnesses.

A vast diversity of biomaterials has been explored and commercialized in ophthalmic drug delivery systems, mostly aiming to circumvent FBR. Conversely, with a deeper dive into the ocular immune response and biomaterial interplay and dissections, we can better understand the active role of biomaterials, and derive potential therapeutic approaches in ocular regenerative medicine and immune interventions. This can be achieved by targeting the FBR cascade, in terms of the distribution and timing of the key immune cellular and molecular players, using biomaterials and delivery devices. Moreover, learning from the natural defense mechanism against pathogens, the biomaterial polymer structure’s repeating units, size, hydrophobicity, and patterning can be fine-tuned to mitigate or engage innate immune cell activation (Kakizawa et al., 2017). In the next section, we discuss several biomaterial systems, such as implants and injectable depots, nano/micro particles, and hybrid systems, as well as their physiochemical requirements for directing ocular innate immune cells to the desired anti- or pro-inflammatory phenotypes.

### 3.1 Implants and injectable depots

FBR is inevitable with the introduction of ocular implants and injectable depots. In ocular therapy, biomaterials can be used to implant cells, create therapeutic implants, and create injectable depots. During the initial stage of FBR, the acute inflammatory response generates pro-inflammatory cytokines to promote inflammatory cell infiltration, extracellular matrix (ECM), vascular remodeling, and perfusion. This can lead to an increase in ocular pressure, oxidative stress, ischemia, stress, and retinal illnesses, all of which are detrimental to the ocular structure and function. The chronic inflammation process often leads to deposition of the collagenous matrix surrounding the implants, causing rejection. Particularly for the intraocular lens (IOL), intravitreal drug depots, retinal prostheses, other long-term ocular implants, and fibrotic encapsulation not only result in device wastage but also vision compromise (Anderson et al., 2008; Veiseh and Vegas, 2019). Therefore, the timing and dynamics of FBR are important targets for biomaterial development.

An anti-FBR approach is rather common in ocular applications, especially for extended, controlled delivery platforms and prostheses. Biomaterials and delivery platforms are often used in an “immune stealth” or anti-inflammatory context to inhibit immune recognition and suppress the deposition of proteins and cells; thereby extending the half-life and bioavailability of therapeutic entities. Polyethylene glycol (PEG) is one of the most popular stealth materials in suppressing non-specific protein deposition on hydrophilic polymer coated devices. In some applications, it is useful for the biomaterial to mimic the ECM of the tissue, in terms of biochemical and biomechanical properties, to suppress the immune activation. The major compositions of ocular ECM are hyaluronic acid and collagens. For this ECM-mimicking purpose, the use of ECM-derived components and analogs, such as hyaluronic acid, fibrin, and collagen, as well as decellularized tissue, has been extensively explored (Rowley et al., 2019). These materials are commonly used in the development of intravitreal implants and corneal grafts. Moreover, ECM-derived peptides, including arginyl-glycyl-aspartic acid (RGD), matrix metalloproteinase (MMP)-sensitive peptides, or leukocyte-associated immunoglobulin-like receptor-1 (LAIR-1) ligand, are also incorporated on synthetic materials to improve the biocompatibility of the implants (Rowley et al., 2019). The design parameters of biomaterials for the anti-FBR approach is further discussed in the following section.

#### 3.1.1 Hydrogels and polymeric depots

Hydrogels are widely used as delivery devices and supporting scaffolds in the ocular field. Hydrogels are 3D crosslinked hydrophilic polymers. They can hold small hydrophilic molecular drugs, and bioactive molecules such as proteins and nucleic acids. They can protect labile cargoes from tissue clearance and enzymatic degradation. Controllable hydrogel architecture design and degradability can help provide spatial and temporally controlled release of cargoes. More importantly, hydrogels also possess mechanical and optical characteristics that are compatible with those of the eyes.

For anterior ocular treatments, hydrogels are commonly used in the preparation of artificial tears, corneal regeneration, and IOL fabrication to provide structural support. Non-etheless, implantation sites such as the ocular surface and epithelial and stromal regions are populated with innate immune cells. They are sensitive to environmental anomalies triggered by the implants, eye drops, and injectables, subsequently inducing a cascade inflammatory response. Indeed, corneal haze and posterior capsular opacification (PCO) are common post-surgical complications in artificial corneal and IOL transplants, respectively.

On the other hand, for the ocular posterior, hydrogels can be used as vitreous substitutes to regulate intraocular pressure and structures. Delivery depots can protect laden drugs and therapeutic cells while providing controlled release of the therapeutic molecules directly to the diseased sites, circumventing the blood-retinal barrier. With appropriate biomaterial selection, FBR can be suppressed. For instance, a single intravitreal injection of *in-situ* crosslinkable hyaluronate (HA)/dextran hydrogel, which was designed to provide sustained release of anti-VEGF for 6-months, showed no inflammation of the ocular structures (Yu et al., 2015; Yu et al., 2019). In addition, the prolonged presence of thermosensitive methoxy-poly (ethylene glycol)-block-poly (lactic-co-glycolic acid) (mPEG-PLGA-BOX) hydrogel in the vitreal region, providing extended release of anti-VEGF for 42 days, also avoided inflammation in the posterior ocular region (Hu et al., 2019). However, another study that adopted PolyActive™, degradable PEG-polybutylene terephthalate-based (PEG-PBT-based) microparticles, to deliver anti-VEGF, observed inflammation at both acute initial and later stages of the treatment, suggesting that degradation products from the microparticles could trigger immunogenic responses in the eye (Adamson et al., 2016). The co-delivery of immunosuppressive signals also inhibited FBR against the long-term device. For instance, the commercial intravitreal injectable Ozurdex® is a degradable poly (lactide-co-glycolide) (PLGA) matrix for extended release of dexamethasone to circumvent FBR and actively suppress inflammatory events of uveitis.

Therefore, it is crucial to maintain ocular immune tolerance, that is, anti-inflammatory and anti-FBR responses, in order to improve the lifetime and optimal performance of the delivery device (Allyn et al., 2022). The general design strategies of implants and injectables often aim to 1) reduce non-specific protein and cellular adhesion or design anti-fouling properties to circumvent FBR, 2) introduce a delivery system with tissue-matching properties, and 3) mask receptors associated with pro-inflammatory pathway activation for suppressing innate immune activations. In the first approach, biomaterials and delivery platforms are often used in an “immune stealth” or anti-inflammatory context to inhibit immune recognition and to suppress the deposition of proteins and cells, thereby extending the half-life and bioavailability of therapeutic entities. PEG is amongst the most popular stealth material in suppressing non-specific protein deposition on hydrophilic polymer-coated devices.

#### 3.1.2 Encapsulated cell implants

##### 3.1.2.1 Immunomodulatory cells

For decades, it has been demonstrated that mesenchymal stem cells (MSCs) are potential immunomodulators of the innate and adaptive immune systems (by inhibiting Th1 and Th17 and inducing the Treg and M2 macrophage phases) for various ocular inflammatory disorders such as corneal angiogenesis, neovascularization, autoimmune uveitis, and autoimmune DED (Lee et al., 2015; Song et al., 2018; Oh and Lee, 2021; Li Y. et al., 2022).

For targeted delivery to the eye, MSCs can be injected naked or encapsulated in a polymeric scaffold such as hydrogels to promote ocular tissue regeneration or suppress degeneration. The hydrogels can serve as a supporting matrix to separate the cells’ cargo from the host tissue. In addition, they will support cell growth and functions, while the meshwork can facilitate sufficient exchange of materials between implanted cells and their host tissues, ensuring that laden cells are functioning properly. The choice of biomaterial is critical in the second strategy, to achieve immunomodulatory and biocompatibility goals using different administration routes such as topical or contact lenses or injectable scaffolds.

##### 3.1.2.2 Implants for encapsulating engineered cells

Based on previous works on several anti-inflammatory applications using MSCs and bandage contact lenses, the amniotic membrane (AM) is the most employed scaffold in the topical administration of MSCs in ocular surface therapy (Abu-Ain and Webber, 2010). AM exhibited remarkable anti-inflammatory and immunomodulatory effects (Parolini et al., 2009; Orozco Morales et al., 2019; Beeken et al., 2021). However, due to donor variability and the danger of disease transmission, this procedure lacks standard safety regulations. Synthetic and natural hydrogels can be employed as substitutes for AMs in this application. For instance, fibrin gel was used in a study as the carrier of rabbit MSCs and was transplanted on the surface of damaged rabbit cornea; it can be differentiated to corneal epithelial cells and inhibited inflammation in the area (Gu et al., 2009). Another scaffold that has been used is polylactic acid (PLA) nanofibers for seeding bone marrow-derived MSCs (BM-MSCs), adipose tissue-derived MSCs (Ad-MSCs), or limbal epithelial stem cells (LSCs) for the treatment of corneal optical properties after alkali burns that precede the inflammatory response (Cejka et al., 2016). BM-MSCs nanofibers and LSCs nanofibers are capable of suppressing corneal inflammation and neovascularization by suppressing MMP9, iNOS, and VEGF in the cornea. Polyamide 6/12 nanofiber scaffold (Zajicova et al., 2010) is another example of using hydrogels with MSCs in ocular surface inflammations; it significantly reduced the local inflammatory response in various ocular surface injuries.

Counterintuitively, the inflammatory response triggered by biomaterials can be leveraged to accelerate tissue regeneration, involving MSC and LSC. Polysaccharide has been shown to accelerate the tissue repair process by stimulating the inflammatory phase, with increased activation of macrophages, infiltrating cells, and fibroblasts (Matica et al., 2019). In this way, the acute inflammatory phase can be soon taken over by the proliferative phase to facilitate rapid re-epithelization and wound closure. The timing and dynamics of the inflammatory response are important targets for biomaterial development. Particularly, at the early stage of inflammation, intervention using biomaterials can promote pro-healing responses, predominantly for the delivery of therapeutic entities for ocular tissue regeneration.

Besides the use of MSC-encapsulated implants as an immunomodulatory treatment, there is another intriguing cellular therapeutic strategy that has been tested in clinical trials for eye disorders. In this strategy, cells are genetically transfected before encapsulation, and by then loading cells inside the polymeric implant, the engineered cells are capable of producing the targeted protein for a prolonged time (Zhang et al., 2011; Wong et al., 2017; Belhaj et al., 2020). The polymeric implant is permeable, allowing the therapeutic drug to diffuse while protecting cells from the host’s immunological responses. As a result, the selection of biomaterial as a protective carrier is critical to avoid inducing an unwanted immune response, or, even one step better, inducing an anti-inflammatory response. Neurotech is a pioneer in this technology, having developed the NT-501 device to provide sustained delivery of ciliary neurotrophic factor (CNTF; a growth factor shown to decrease photoreceptor degeneration in RP animal models) to treat retinal degenerative diseases such as RP, AMD, and glaucoma (Zhang et al., 2011; Kauper et al., 2012a; Kauper et al., 2012b; Chew et al., 2015).

Early clinical trials employed polymeric matrix-based systems, such as NT-501 (Renexus®), which is composed of a semi-permeable, non-biodegradable polysulfone scaffold, with polyethylene terephthalate as an internal matrix, to support CNTF-producing ARPE-19 cells. However, this cell-encapsulating device measured 1 mm in diameter and 6 mm in length, and requires surgical insertion in the vitreal region. NT-501 is currently under stage 2 clinical trials for glaucoma, and no device-related adverse events have been reported so far (Zhang et al., 2011; Wong et al., 2017). This method may be used for long-term delivery of different proteins and polypeptides such as VEGF-antagonist, inhibitory domain of factor H (complement receptor 2 and factor H (CR2-fH)) as a complement inhibitor of CNV, or anti-inflammatory cytokine target to suppress inflammation in inflammatory diseases (Adamson et al., 2016; Belhaj et al., 2020).

To improve patient compliance and ease of use, several hydrogel alternatives have been proposed, using a less-invasive injection procedure in encapsulated cell therapy. Immune-compatible materials, such as alginate and collagen-based hydrogels have been developed to deliver cells. Studies demonstrated minimal host-cells attachment on the hydrogel surface, the presence of living cell colonies, and the generation of active biomolecules over an extended number of days of implantation (Wikström et al., 2008; Wong et al., 2017; Belhaj et al., 2020). For instance, in one animal study, ARPE-19 cells were transfected to express the gene of choice and encapsulated in alginate polymer using a microencapsulation method coupled with electrospray to generate an encapsulated cell alginate capsule. The size of the capsule was controlled by the alginate concentration and the voltage of the electrospray. The results showed that a size of 150 μ l was more suitable for intravitreal injection, and that this method can be used for long-term delivery (around 6 weeks) of complement inhibitors for AMD treatment. The authors’ technique was reported to be appropriate for animal models as it lacks the stability of alginate, which prevents it from being destroyed during injection (Wikström et al., 2008; Belhaj et al., 2020). In future, it may be worthwhile to study the immunomodulatory effect of biomaterials as implants for improving the functionalities of these encapsulated cell implants.

#### 3.1.3 Implants and injectable depots design parameters

##### 3.1.3.1 Mechanical strength

Ocular tissues exhibit different mechanical strengths, with the cornea at 3.8 MPa, IOL at 2.5–6 MPa, and vitreous 20–50 Pa. Tissue stiffness is subject to differences in species, age, and disease states (Nickerson et al., 2004; Formisano et al., 2021). Corneas and IOLs undergo substantial mechanical loading and stretch as part of their normal functions in maintaining ocular pressure and visual acuity. Resident innate immune cells have been exposed and habituated to these mechanical stimuli during physiological conditions (Liu and Li, 2021; Du et al., 2022). However, during diseased conditions, such as during glaucoma, the change in intraocular pressure and mechanical environment may induce pro-inflammatory activation of resident immune cells and may exacerbate disease progression. In fact, mechanical sensors, transient receptor potential vanilloid (TRPV), pannexin-1, and P2X7 can be found on retinal ganglion cells (RGCs) and glial (Müller) cells in posterior ocular structures (Križaj et al., 2014). The activation of mechanosensors may result in the release of pro-inflammatory mediators, such as IL-1β, which can in turn promote the activation of nuclear factor kappa B (NF-κB) in microglia. On the other hand, the corneal epithelium, which comprises the major ocular anterior surface, is able to sense shear stress. Abnormalities in mechanical properties can trigger the activation of resident innate immune cells (Liu and Li, 2021). Pathological conditions may result in dry eye disease.

Therefore, hydrogel stiffness should match tissue stiffness depending on the implantation site, to help maintain ocular structures and immune responses. The mechanical strength of hydrogels can be tuned by changing the polymer concentration, molecular weight of polymers, and crosslinking densities, as well as by incorporating nanomaterials and composites. For instance, in dry eye symptom relief, our group reported a HA-based soft hydrogel, which matched the mechanical properties of the tear film and provided surface lubrication for an extended period of time, without frequent corneal instillation. Moreover, when combined with cyclosporine, an immunosuppressant, the treatment regime significantly improved the symptoms of dry eye diseases in canine clinical studies, in comparison to cyclosporine treatment alone (Yu et al., 2021). It was reported that hard methacrylate-gelatin (GelMA) hydrogel, with a measured strength of 29 kPa, can induce M1 differentiation of macrophages *in vitro* and severe FBR responses *in vivo*, in contrast to softer alternatives with strengths of 2 and 10 kPa (Zhuang et al., 2020). A similar trend was observed in the THP-1 macrophage line culture on collagen-coated polyacrylamide gels, with higher M1 polarization on hard gels (323 kPa) than softer gels (11 kPa and 88 kPa). It was also demonstrated that the hydrogel stiffness modulated macrophage migration behavior, with a higher spreading area and slower movement on the stiffer gel. Moreover, it was reported that macrophages exhibit lower phagocytic ability on a stiffer gel (Sridharan et al., 2019; Li and Bratlie, 2021).

Soft hydrogels are generally preferred in ocular applications and are favored in the structural design of the ocular surface and intravitreal implants, with minimal adverse immune responses. However, tissue engineering for corneas and IOLs requires hydrogel scaffolds with much higher mechanical strengths to withstand ocular structural changes and functions. To accommodate these mechanical needs, other design modifications, such as surface hydrophobicity and topography are pursued to suppress immune activation.

##### 3.1.3.2 Surface chemistry: Charge and hydrophobicity

In the case of cornea and IOL implantations, hydrophobic materials are generally preferred in order to resist swelling, lens epithelial cell engagement, and migration from the peripheral to the visual region (Zhao et al., 2017). Non-etheless, PCO and corneal haze are common among patients after surgery, both of which are characterized by the FBR of prostheses, resulting in secondary visual impairment (Pintwala et al., 2014; Tamura et al., 2017). Indeed, it is generally accepted that a hydrophobic surface can promote non-specific protein deposition and initiation of FBR. Hunter et al. (1981) were the first researchers who worked on the adjuvant efficiency of the polymer backbone structure. It has been shown that different inflammatory and immune responses are stimulated based on the physiochemical properties (hydrophile-lipophile balance) of the biomaterial and that the hydrophobic part of the molecule can stimulate the innate immune cells and cause inherent immunogenicity by PAMP or DAMP (Seong and Matzinger, 2004; Andorko and Jewell, 2017). The process is characterized by the fusion of macrophages to form giant body cells to facilitate the clearance of foreign materials. The cells also secrete immunomodulatory cytokines such as TNF-α, IL-1, and TGF-β which can further promote nearby fibroblasts to secrete fibrinogen that surrounds the prostheses to isolate them from the host tissue environment.

Common strategies to bypass FBR include modifying the surface charge and hydrophobicity of implants. Hydrophilic PEG and anionic and zwitterionic polymers are often selected as coating materials for such purposes. There are several ways to modify the implant surface, and these generally involve physical or chemical methods, as discussed in a previous review (Song et al., 2020). The physical methods include electrospinning/spraying, spin coating, dip coating, and layer-by-layer (LbL) polymer assembly; chemical methods generally involve radical polymerization, 3-aminopropyltriethoxylsilane (APTES) and glutaraldehyde, carbodiimide coupling, or “click” chemistry. Indeed, studies on surface modification of the commercial artificial corneas Keraklear, Korea Seoul-type, and T-style keratoprostheses demonstrated that coatings of hydrophobic poly (methyl methacrylate) (PMMA) or poly (2-hydroxyethyl methacrylate) (PHEMA) with PEG, a hydrophilic polymer, can effectively resist immune cell engagement and suppress the secretion of pro-inflammatory cytokines (Kim et al., 2002; Xiang et al., 2015; Farid et al., 2020). As well as PEG, implant surface coatings with zwitterionic polymer brushes, such as phosphorylcholine and sulfobetaine, have also been utilized, and results demonstrated superior capabilities in suppressing FBR (Han et al., 2017; Wang et al., 2021). Other natural hydrophilic polymers, such as anionic HA, have been employed together with lysozyme in PMMA surface coating; they exhibited anti-bacterial properties and reduced the adhesion of cells and bacteria in *in vitro* culture (Wang et al., 2014). Moreover, a silicone-based hydrogel with a surface LbL assembly of alginate and chitosan was developed to achieve sustained release of the immunosuppressant diclofenac to suppress post-surgical complications related to IOLs (Silva et al., 2016). The hydrogel surface charge can also influence the treatment outcomes of several posterior ocular diseases. In the case of glaucoma, the suprachoroidal injection of HA hydrogel produced a shorter retention time and showed minor inflammation and fibrosis at the injection site. However, the application of the zwitterionic hydrogel polycarboxybetaine significantly suppressed inflammation and prolonged the reduction of intraocular pressure (IOP) *via* suprachoroidal space expansion (Hao et al., 2022).

##### 3.1.3.3 Surface topography and porosity

On top of the choice of hydrophilic or hydrophobic material chemistry, the surface roughness or topography of a hydrogel can also affect the wetting state, protein adsorption, and cell interactions. There is increasing evidence that hydrogel surface topography can modulate cell behaviors such as adhesion, migration, proliferation, and differentiation. During fabrication, hydrogel surface patterning can be achieved, up to micron and sub-micron resolutions, with the appropriate choice of materials and pattern design to counter the swelling behavior while preserving the pattern fidelity. The fabrication methods often include casting, nanoimprinting, 3D printing, electrospinning, multiphoton patterning, lithographic patterning, and swelling-induced patterning (Cui et al., 2021).

Surface patterning of hydrogels can influence the wettability of materials. In the case of micropillar array design, the spacing and height interfere with the water contact angle and protein adsorption, as well as cell-material interactions. A high throughput screening approach using a diverse library of 2,176 micropatterns was recently developed to study the relationship between monocyte-derived macrophages and topography. The study revealed that diameters of 5–10 μm favored macrophage attachment, and smaller, denser micropillars pattern can instruct M2 phenotype polarization (Vassey et al., 2020). Another study also reported that micropillars with 2 μm spacing and 4.5 μm in height are able to resist silicone hydrogel wettability as well as protein and cell adhesion (Papenburg et al., 2010). On the other hand, PVA with 2 μm gratings and a concave lens topography of 1.8 μm can promote endothelial cell adhesion on PVA hydrogel, compared with wider (10 μm) gratings, pillars, and convex lens textures (McWhorter et al., 2013). In general, surface texture design often aims to mimic niche tissue topographical features to better direct cell behaviors for optimal delivery device performance and therapeutic efficacy. IOLs with surface micropatterning are able to resist PCO. The presence of micron-sized isotropic elements arranged in a symmetric and regular pattern can resist cell adhesion and migration. It was suggested that the texture interfered with the formation of focal adhesion within cells, which hampers cell adhesion and spreading. Indeed, the study demonstrated that ridge(R)/grooves(G) patterns with sizes comparable to cells, e.g., R5G10 with a gradient spacing, can also significantly suppress fibroblast cell adhesion and promote directional cell migration on the culture surface (Kwon et al., 2017).

Conversely, anisotropic geometries, such as gratings, can promote cell differentiation. It was observed that macrophages in the pro-healing M2 state exhibit extended or oblong morphology, in comparison to M1. Several studies demonstrated that by controlling macrophage morphology using micropatterning methods, macrophages can be polarized into the M2 state without the addition of cytokines. The cells cultured on confined micropatterned fibronectin lines 20 μm in width were able to produce higher levels of IL-4 and IL-13 and resisted the effect of M1-inducing LPS stimulus (McWhorter et al., 2013). In addition, a honeycomb-like surface pattern 90 nm in size can also promote M2 differentiation, with highest expressions observed for CD206, IL-4, IL-10, and growth factors, which support tissue regeneration. It was suggested that the honeycomb-like texture can promote filopodia formation, which is associated with RhoA/ROCK signaling pathways, and can induce M2 polarization (Zhu et al., 2021). Moreover, a study reported that fibrous meshes of gelatin hydrogel nanofibers mimicking ECM promoted macrophage adhesion and differentiation into M2 phenotypes, with upregulation of CD206 expression and downregulation of IL-1β and IL-8 in *in vitro* culture (Taskin et al., 2021).

The porosity of hydrogel implants can also be tuned to modulate immune cell behaviors. Under physiological conditions, cells reside in porous ECM which provides optimal mechanical cues and spaces for biochemical cue exchange and cell-cell communications. In pathological challenges, ECM polymer backbones can support immune cell infiltration to wound sites. The design of microporous hydrogels aims to promote immune cell infiltration for local antigen delivery or immunomodulation. This can be achieved by introducing degradable porogens during hydrogel fabrication. A study reported an alginate-based porous hydrogel system incorporating rapidly degradable alginate beads into a non-degrading bulk alginate hydrogel. The authors further adjusted the porosity of the hydrogel by adjusting the percentage of porogens. The study revealed that a system with 50% porogen significantly promoted DC infiltration and maintained the immature phenotype, compared with 25% and 70% porosity (Verbeke et al., 2015).

The size of pores can also be adjusted to protect cargoes from immune cell-mediated clearance while preserving materials exchange between the carrier and host tissue environments. For instance, nanopores can be introduced to cell-laden hydrogels to ensure that the cargo survives and functions. Indeed, an alginate hydrogel with a pore size of 600 nm was developed to encapsulate mesenchymal stem cells and was able to resist pro-inflammatory immune cell infiltration (Moshaverinia et al., 2015). Such hydrogel design could promote stem cell survival and the maintenance of phenotypes to support tissue regeneration.

##### 3.1.3.4 Degradability

Biodegradable hydrogels have received increasing attention for the design of drug delivery systems for spatiotemporally controlled release, protection from physiological degradation and clearance, and improved patient compliance. Hydrogel drug depots can be injected into the vitreal space to provide extended release and to improve the bioavailability of therapeutics for posterior ocular disease treatment. Drug depots are often designed to match the optical and mechanical properties of tissue sites to preserve visual functions. Moreover, degradable drug depots are more favored in intravitreal injections to circumvent the invasive surgical retrieval of non-degradable implants from the eye.

Generally, depending on the hydrogel design, the depots can undergo different types of biodegradation mechanisms, namely, solubilization, chemical hydrolysis, and enzymatic degradation. More importantly, the drug release kinetics can be controlled by the hydrogel degradation rate. There are several strategies that have been developed to regulate hydrogel degradation kinetics, which involve physical and chemical factors. For instance, physical interventions include changing the polymer concentration, the molecular weights of hydrogel precursors, hydrogel size, architecture, and microstructure. On the other hand, chemical methods include introducing functional groups to modify polymer charge or hydrophobicity, the nature of crosslinkers, and crosslinking density (Kong et al., 2004; Jain et al., 2017; Lau et al., 2021).

**FIGURE 3:**
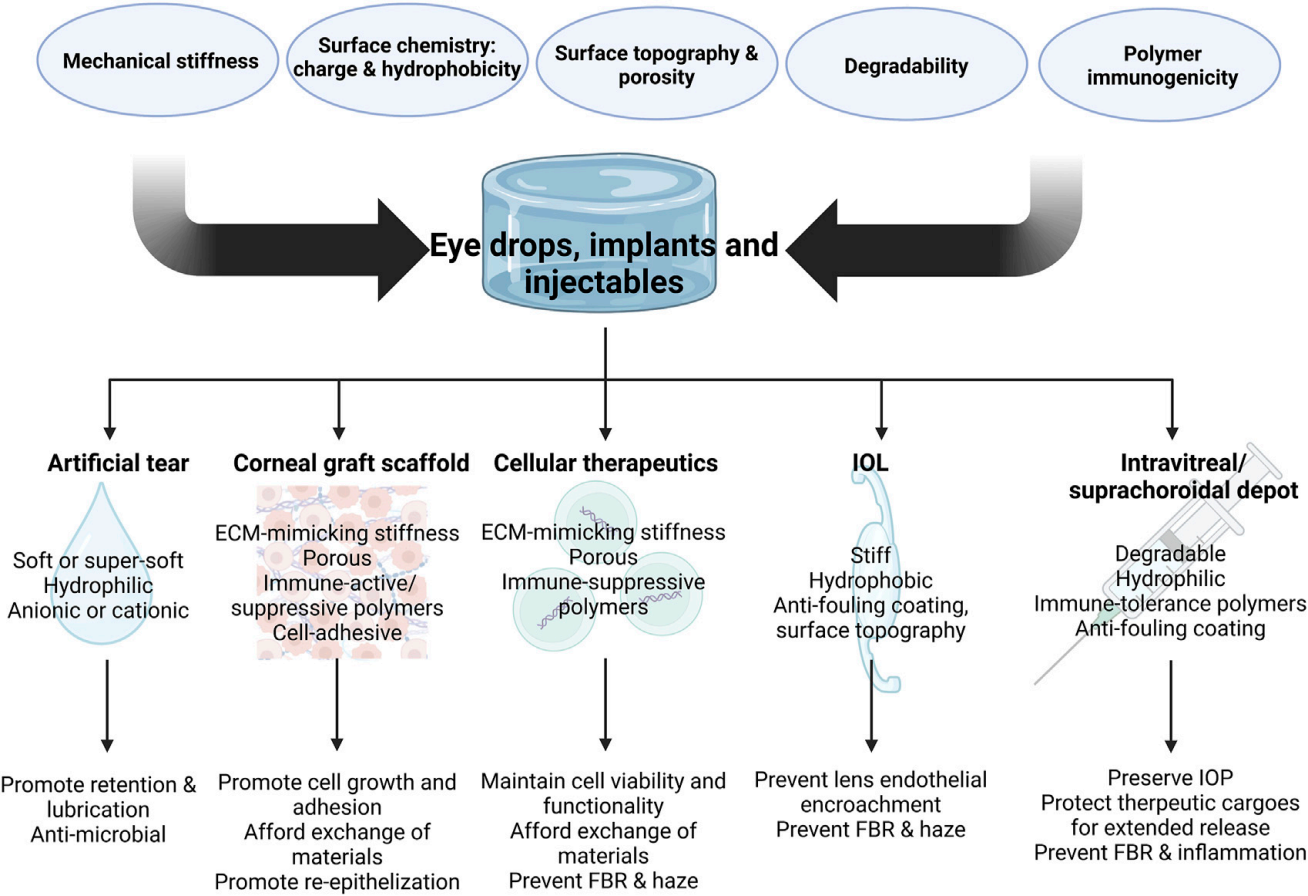
Design parameters of implants and injectable depots for various ocular applications and key features for desired therapeutic outcomes (Created in BioRender.com).

Non-etheless, the long-term presence of hydrogel depots in the intravitreal space is often associated with FBR, and the design of degradable depot formulations can induce chronic inflammation, thereby hampering the immune compatibility of the delivery device. In fact, an undesirable immune response has been observed in vitreous substitutes that consisted of HA and PEG. In the case of PEG, despite being known for its excellent anti-fouling properties in bypassing FBR, its *in vivo* degradation has been shown to induce the generation of anti-PEG antibodies and local inflammatory responses (Reid et al., 2015; Chen et al., 2021). Moreover, for HA hydrogels, the choice of molecular weights during preparation is crucial in regulating its immunomodulating properties. Generally, higher HA molecular weights (>500 kDa) are preferred, as they can induce anti-inflammatory responses by promoting macrophage M2 polarization and tolerogenic dendritic cell induction. Our group observed that a higher HA molecular weight of 670 kDa could avoid inflammatory responses when the polymers were used to coat lipid-based nanoparticles to improve intravitreal retention in rabbit eyes. Non-etheless, with the use of small HA molecular weights such as 36 kDa and 120 kDa, inflammation is observed in posterior ocular structures (Xiaonan, 2020). The underlying mechanism is due to HA interactions with PAMP/DAMP receptors on innate immune cells, in which the high molecular weight biopolymer exerts competitive engagement of TLR2 and/or TLR4 receptors of the immune cells, resulting in signaling inhibition of pro-inflammatory pathways governed by Myeloid differentiation primary response 88 (MyD88) and NF-κB. The smaller molecular weight alternatives, on the other, hand can act as TLR agonists (Østerholt et al., 2011; Rayahin et al., 2015; Gebe et al., 2017). A recent study used zwitterionic hydrogel composed of polycarboxybetaine and demonstrated its superior anti-fouling ability against proteins and cells. The material was then used as a vitreous substitute in a rabbit model, with no visible adverse effect (He et al., 2021) (Figure 3).

### 3.2 Nano/micro particles

For years, nano/micro particles have been used as common drug delivery systems in the eye and could be a potential drug delivery carrier for ophthalmic applications. Nanoparticles (NPs) are considered to have a size under 1000 nm and microparticles (MPs) between 1 micron and 1 mm. MPs can be used as microcapsules (a core drug with a polymeric layer surrounding it) or microspheres (drugs dispersed throughout the polymer matrix), based on their structure (Herrero-Vanrell et al., 2014). Microcapsules are also referred to as ocular implants and these have already been covered in the section on implants (Zimmer and Kreuter, 1995; Adamson et al., 2016).

MPs are usually used in ocular applications for prolonged delivery (at least 1 week) of proteins, peptides, and biomolecules. PLGA is the most commonly used polymer in this area (Cohen et al., 1991; Carrasquillo et al., 2003; Gavini et al., 2004; Mandal et al., 2018). For instance, Gavini et al. (2004) developed vancomycin-encapsulated PLGA microspheres for topical ocular delivery, and showed a high concentration of peptide drug in the rabbit aqueous humor just after 3 h of topical administration. Albumin, gelatin, PLA, polyanhydrides (Ron et al., 1993), and cyclodextrins (Davis and Brewster, 2004) are other biodegradable polymers that are used as microspheres. For the sustained release of pilocarpine, which is used to lower intraocular pressure, Rathod et al. developed pilocarpine-loaded egg albumin microspheres as an eye drop (Rathod and Deshpande, 2008). Another work on the transscleral delivery of PLGA microspheres encapsulated with pegaptanib sodium (an RNA aptamer (EYE001) that inhibits VEGF) showed sustained delivery of EYE001 over 20 days on the surface of the sclera to treat choroidal and retinal diseases such as wet AMD (Carrasquillo et al., 2003). The administration route and the type of polymer significantly alter the inflammatory response of innate immune cells in microsphere ocular delivery systems. For instance, it has been demonstrated that intravitreal injection of PLA and PLGA microparticles may increase inflammatory responses due to their propensity to aggregate, making them more suitable for use as depots for the simultaneous delivery of several biomolecules loaded into nanoparticles (Herrero-Vanrell et al., 2014).

It has been demonstrated in the literature that NPs are favored over MPs in circumstances of prolonged drug delivery and targeted delivery to deeper ocular layers (Zimmer and Kreuter, 1995; Andrés-Guerrero et al., 2015; Huang and Chau, 2019a). The use of NPs in the treatment of ocular disorders is a promising new tactic that may pave the way for more successful ocular therapy and penetration for targeting posterior parts of the eye. They may target the cornea, retina, and choroid, and exhibit several advantages for use in the treatment of eye disorders. These systems are capable of transporting various cargoes. For instance, the use of NPs may make it easier and more effective to distribute hydrophobic anti-inflammatory drugs such as corticosteroids and unstable nucleic acids such as mRNA. Furthermore, since they can pass through intricate ocular barriers such as ocular-retinal barriers and blood-retinal barriers of the eye, their delivery route through difficult-to-access areas of the eye is facilitated. Additionally, they sustain the long-term release of immunomodulatory medications for chronic ocular inflammatory conditions (Diebold and Calonge, 2010; Huang and Chau, 2019a; Tang et al., 2022). Various nano/microparticles that have been employed in immunomodulatory ocular applications are listed in Table 1 as a summary. Smart biomaterials, on the other hand, may be created to target specific tissue locations by employing optical, electrical, or mechanical targeting properties. They have several uses in biological imaging, targeting, smart drug delivery systems, and anti-inflammatory eye treatments (Ren et al., 2019; Luo et al., 2020).

**TABLE 1:** Summary of different micro/nano particles used in the immunomodulation of ocular disorders.

Material	Physicochemical properties	Anti-inflammatory cargo	Ocular disease	Anti-inflammatory agent	Ref.
Pegaptanib sodium-PLGA microsphere	Size 14–16 μ m	EYE001	Choroidal and retinal diseases (wet AMD)	EYE001 RNA to inhibit VEGF	Carrasquillo et al. (2003)
Pilocarpine-egg albumin microsphere eye drop	Size 1–12 μ m	Pilocarpine	IOP in eye	Pilocarpine to reduce IOP	Rathod and Deshpande (2008)
Vancomycin-PLGA microsphere as eye drop	Size ∼11 μ m	Vancomycin	Ocular infections	Vancomycin as peptide antibiotic	Gavini et al. (2004)
Dexamethasone sodium phosphate-Zn-PLGA nanoparticle (DSP-Zn-NP)	Size 210 nm, near neutral surface charge (−9 mV), hydrophobic	Dexamethasone	Uveitis by EAU	Pluronic F127 surface coating and corticosteroid drug	Luo et al. (2019)
Polymeric implant (PVA and silicone)	Size 2 mm, long cylinders, 300 μm in diameter	Fluocinolone acetonide (FA)	Retinal degeneration	FA as corticosteroid drug	Glybina et al. (2009), Glybina et al. (2010)
Rapamycin-loaded PCL-micelles	Size 40nm, sphere, surface charge of −0.89 mV	Rapamycin	EAU	Rapamycin as immunosuppressant drug	Wu et al. (2016)
HA-coated PLGA nanoparticle	Size 173–200 nm, negative surface charge, hydrophilicity increased by using HA	Lutein	AMD	Lutein as antioxidant drug and HA coating	Chittasupho et al. (2019)
Double-headed polyester nanoparticles using gambogic acid (GA)–coupled PLGA (PLGA- ${GA}_{2}$ -CUR)	Size 250 nm, double-headed nanoparticle, hydrophobic	Curcumin (CUR)	Acute intraocular inflammation by EAU	Curcumin as anti-inflammatory drug	Ganugula et al. (2020)
DuraSite/synthetic polymer of cross-linked PAA (Bromfenac)	Adjusted to a viscosity of 1100–1900 cP, pH 6–6.6, osmolality 260–340 mOsm/kg, suitable for dispensing in the eye	1% azithromycin or 0.6% besifloxacin	Post cataract surgery inflammation and pain	Antibiotic drugs	Bowman et al. (2009), Trattler and Hosseini (2017)
					NCT01576952
Dexamethasone-PAMAM dendrimer conjugates (DEX/PAMAM)	Size ∼145 nm, surface charge ∼ −50 mV	Dexamethasone	Ocular diseases affecting retina such as AMD, DR, and glaucoma	Dexamethasone as corticosteroid drug	Yavuz et al. (2016)
Hydroxyl-terminated PAMAM dendrimer-drug conjugate nanodevices	Size ∼3–10 nm, PMMA with −OH terminal groups, non-cytotoxic	FA	Retinal neuroinflammation in AMD and retinitis pigmentosa	FA as corticosteroid drug	Iezzi et al. (2012)
Pilocarpine-loaded hCe Chitosan/ZM nanoparticle	Size less than 70 nm, positive surface charge of 5–20 mV	Pilocarpine	Glaucoma	Pilocarpine drug to reduce IOP and cerium oxide nanoparticles with anti-inflammatory effects	Luo et al. (2020)
Lipoidal–chitosan–poly (ε-caprolactone) nano system (DSPC-chit-PCL NS)	Size ∼140.7 nm, surface charge of ∼ +55 mV	Indomethacin	Inflammatory disorder in the posterior part of the eye	Use of DSPC lipid and indomethacin drug	du Toit et al. (2013)
Betamethasone phosphate/PEG-PLA nanoparticle	Size ∼120 nm	Betamethasone phosphate	Uveitis by EAU	Betamethasone as steroid drug and use of PEG surface coating	Sakai et al. (2006), Sakai et al. (2011)
Gold nanoparticle (AuNP)	Size ∼25 nm	No drug	LPS-induced uveitis model	Inherent anti-inflammatory properties of AuNP by downregulating the TLR4 and NF-κB pathways	Pereira et al. (2012)
VIP-loaded pegylated liposome	Size 300–600 nm	VIP	EAU	VIP as immunosuppressant	Lajavardi et al. (2007)
Cy A-loaded hyaluronic acid-coated PCL/benzalkonium chloride (BKC) nanospheres	Size over 200–300 nm	Cy A	Immune-mediated corneal disease	HA reduced the BKC cytotoxicity and Cy A as inhibitor of IL-2 proinflammatory cytokine	Yenice et al. (2008)
siRNA targeting VEGF nanoball (PEI/HA)	Size 260 nm, surface charge of −41 mV	siRNA anti-VEGF	Ocular neovascularization	siRNA to inhibit the VEGF expression	Ryoo et al. (2017)
K5 plasmid-loaded PLGA/chitosan NP	Size 260 nm, surface charge of 8.4 mV	Expression plasmid of plasminogen Kringle 5 (K5)	Retinal inflammation	K5 plasmid to downregulate the VEGF expression	Park et al. (2009)
IL10 mRNA-loaded SLN/polymeric ligands of dextran and HA/1%PVA as eye drop	Size between 94 and 348 nm, positive surface charge between 26 and 45 mV	IL10 mRNA	Corneal inflammation	IL10 mRNA as anti-inflammatory cytokine expression agent	Gómez-Aguado et al. (2021)

#### 3.2.1 Nanoparticle design parameters

**FIGURE 4:**
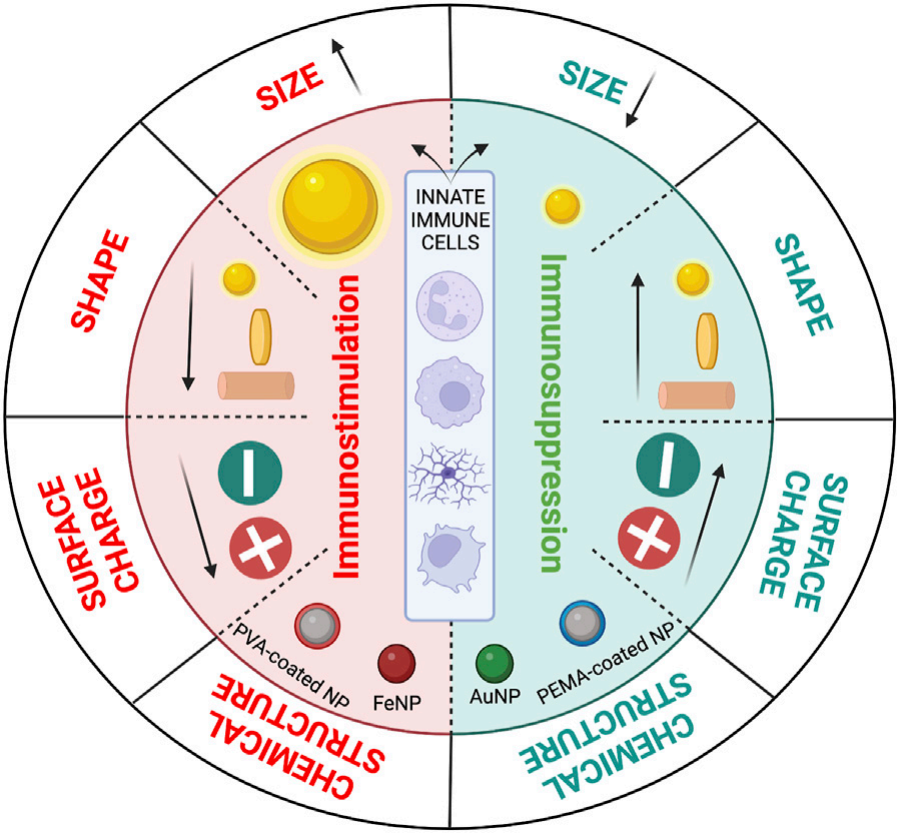
The effect of different nanoparticle design parameters on immune system response. Nanoparticles’ size, shape, surface charge, and chemical structure can lead to immunogenic or immunosuppressive responses from innate immune cells (from top to bottom: neutrophils, macrophages, microglia, and dendritic cells) (Created in BioRender.com).

Immune cells may respond in either a pro- or anti-inflammatory manner depending on the physiochemical characteristics of nanoparticles, including their size, shape, chemical composition, and surface charge (Figure 4). Therefore, by taking these variables into account, nanoparticles can be designed to regulate the immune response. The impact of these parameters is discussed in the following sections.

##### 3.2.1.1 Size

The size of the particle is a key characteristic that drives the innate immune cell response, and as the particle size grows, the innate immune cells’ inflammatory and phagocytosis responses are amplified. The main disadvantages of corneal topical applications are drug bioavailability over time and increasing corneal penetration. Studies have indicated that the nanoparticle size should be less than 200 nm to allow easy uptake via endocytosis in the conjunctiva and cornea. Particle size is directly associated with penetration through the barriers of the cornea. A 180 nm gelatin nanoparticle, used as an eye drop in rabbit eye, for instance, could be retained in the cornea for a long time as a result of being taken up by ocular epithelial cells (Tseng et al., 2013; Soiberman et al., 2017; Tsai et al., 2018; Souto et al., 2019).

The literature also mentions the possibility of injecting positively charged particles with a size range of 50–350 nm into the vitreous body. Notably, for particles bigger than 350 nm, the size impact may be a significant component affecting their distribution, but for particles smaller than 350 nm, the charge effect is a significant parameter (Tsai et al., 2018; Mobaraki et al., 2020). A previous review paper by our group, on intravitreal nanoparticles for retinal delivery (Huang and Chau, 2019a), also noted that nanoparticles, after passing the internal limiting membrane (ILM), are directed to inner layers *via* endocytosis. To determine whether a particle with a particular size and charge may pass through the vitreous and ILM barriers or not, it is crucial to consider the size-cut off for nanoparticles passing through those barriers. Peynshaert et al. provide an in-depth discussion on drug delivery barriers in the posterior segment of the eye (Peynshaert et al., 2018; Peynshaert et al., 2019).

On the other hand, for the treatment of posterior ocular diseases, particles and implants larger than 2 μ m in size remain in the vitreous and are not be able to pass through the retinal and other ocular barriers, similar to earlier reports of intravitreal sustained delivery of fluocinolone acetonide (FA) from a micron-size polymeric implant for over 1 month (Glybina et al., 2009; Glybina et al., 2010; Iezzi et al., 2012). Depending on the type of polymer and administration route used, the particles have the potential to be immunogenic, as previously discussed. Injecting ganciclovir-loaded PLGA microspheres intravitreally, for instance, resulted in their tendency to aggregate by building a depot in the eye, as well as a modest localized FBR response (Herrero-Vanrell et al., 2014). Adamson et al. (2016) also covered the limits of intravitreal injection of anti-VEGF-loaded PolyActive™ microparticles for the treatment of wet AMD. Particle migration from the vitreous to the anterior chamber, a delay in the breakdown of the polymer relative to the release of the payload, and early and late stages of ocular inflammatory response were listed as challenges in using these microparticles in clinical settings (Adamson et al., 2016).

Regarding the immunogenicity and cellular uptake mechanism of smaller nanoparticles, there are still several unanswered problems. Therefore, a crucial factor in identifying whether the innate immune cell interaction pathway is pro- or anti-inflammatory is particle size. In 1998 (D’orazio and Niederkorn, 1998) an intriguing experiment was carried out by D’Orazio et al. The authors investigated the ocular APC response to various antigen types, as well as how the type of antigen affected anterior chamber-associated immune deviation (ACAID), which is a defense mechanism for controlling and avoiding immunologically induced damage. Ovalbumin (OVA) antigen was used as a model antigen. It arrived in two forms: soluble and particulate, and it was passively absorbed on the surface of latex (polystyrene) particles with a size of 460 nm. The authors demonstrated that particulate antigens can stimulate APCs, increasing IL12 production and Th1 activation, whereas soluble antigens activate the ACAID pathway, increasing IL10 production and Th2 regulatory cells (D’Orazio et al., 2001).

##### 3.2.1.2 Chemical structure

Another factor that may affect whether the response of the innate immune system is pro- or anti-inflammatory, as previously mentioned, is the intrinsic chemical structure of the nanoparticle. Natural biomaterials such as gelatin (Vandervoort and Ludwig, 2004; Mathurm and Gilhotra, 2011), chitosan (Marõ´a et al., 2003; Enríquez de Salamanca et al., 2006; Tsukamoto et al., 2013; Li et al., 2009; Aşık et al., 2013), collagen (Luis et al., 2016; Simpson et al., 2021; Song et al., 2021), albumin (Zimmer et al., 1994; Tiwari et al., 2021), alginate (Shafie and Fayek, 2013; Kostenko et al., 2022), and HA (de La Fuente et al., 2008; Yenice et al., 2008; Rayahin et al., 2015; Gebe et al., 2017; Guter and Breunig, 2017; Casey-Power et al., 2022) are frequently used as anti-inflammatory agents in eye treatment because they do not cause an inflammatory response from the innate immune system (Sharma et al., 2016). They come in the form of nanoparticles, nano emulsions, nano capsules, liposomes, and lipid nanoparticles. Although these biomaterials have been used in numerous previous studies as carriers to support drug release, enhance ocular penetration, lengthen ocular retention, and improve ocular bioavailability, future research could focus on the materials’ interaction with ocular innate immune cells to suppress the inflammatory phase of macrophages, without the use of steroid drugs.

Chitosan, a common nanomaterial in ocular research, has been proven in several studies to exhibit minimal immunogenicity for innate immune cells, as previously stated. Chitosan is bioadhesive, can enhance ocular penetration, and does not cause an inflammatory response, according to several experiments (Marõ´a et al., 2003; Enríquez de Salamanca et al., 2006). Furthermore, HA as a nanoparticle or coating of polymeric nanoparticles has shown substantial potential in reprogramming ocular innate immune cells (Yenice et al., 2008; Guter and Breunig, 2017; You et al., 2020). A higher HA molecular weight, as previously noted, may trigger an anti-inflammatory response of innate immune cells (Casey-Power et al., 2022).

On the other hand, PLGA (Casey et al., 2019; Ganugula et al., 2020; Luo et al., 2020), PLA (Sakai et al., 2006; Sakai et al., 2011), poly-caprolactone (PCL) (Yenice et al., 2008; du Toit et al., 2013), polyacrylic acid (PAA) (Trattler and Hosseini, 2017), and poly (amidoamine) (PAMAM) (Iezzi et al., 2012; Yavuz et al., 2016) are the most commonly used synthetic biomaterials as carriers of anti-inflammatory medications in ophthalmic therapy. According to Casey et al. (2019), PLGA and PLA particles have broad-acting mechanisms that inhibit TLR signaling by programming innate immune cells, and they exhibit inherent immunomodulatory properties depending on their physiochemical properties. The authors declared that more research into the inherent immunomodulatory properties of polymer-based particles is necessary since they have the potential to treat a wide range of human disorders *via* abnormal TLR activation (Casey et al., 2019). Similar to natural biomaterials, they come in form of dendrimers, nano capsules, and nano emulsions. Nevertheless, the fact that they degrade *in vivo* raises further questions regarding their immunogenicity, and more research is required to ascertain how different types of materials impact immunity. For instance, in order to prevent the development of experimental autoimmune uveitis (EAU), Wu et al. created PCL-micelles loaded with rapamycin that were 40 nm in size, spherical in form, and with −0.89 mV charge (Wu et al., 2016). Another study aimed to reduce uveitis inflammation by employing PLGA nanoparticles (210 nm) containing dexamethasone (Luo et al., 2019). The cytotoxicity of several materials, including PLGA, PCL, and PEG-PLGA, toward the retinal cell line (ARPE-19) was also tested by Lin et al. The authors found that PEG-PLGA had the lowest cytotoxicity over 6 days, in comparison to the other materials, which exhibited different cytotoxicities (Lin et al., 2016). Another group claimed that surface modification of synthetic polymers reduces their cytotoxicity and subsequent immune responses since HA-coating on PLGA nanoparticles did not exhibit any cytotoxic impact on RPE cells (Chittasupho et al., 2019).

Aside from these two groups, there are other nanomaterials with inherent immunosuppressive functions (anti-inflammatory, anti-angiogenesis, anti-bacterial, and anti-oxidative stress) that have been shown to influence ocular inflammation, including cerium oxide NPs for AMD treatment (Tisi et al., 2019; Luo et al., 2020), gold NPs for uveitis and AMD (Pereira et al., 2012; Singh et al., 2020), and silver NPs for fungal keratitis (Shi et al., 2021). For instance, Pereira et al. (2012) studied the inflammatory response of gold nanoparticles (AuNP) in endotoxin-induced uveitis and showed that the topical use of AuNP reduces inflammation by downregulating the TLR4 and NF- κ B pathways. Another example is cerium oxide nanoparticles (nanoceria), which exhibit inherent immunomodulatory, antioxidant, and anti-inflammatory effects on innate immune cells such as macrophages. These effects can increase the production of anti-inflammatory cytokines while decreasing the release of pro-inflammatory cytokines (Hirst et al., 2009; Schanen et al., 2013; Eitan et al., 2015; Domala et al., 2019; Mitarotonda et al., 2022). Luo et al. (2020) designed antioxidant and anti-inflammatory hollow nanoparticles that deliver anti-glaucoma pilocarpine drugs in a sustained manner for 7 days, as compared to typical eye drops with just 4 h moderate efficacy for glaucoma therapy. The authors chose ceria nanoparticles because of their intrinsic antioxidant, anti-inflammatory, and anti-angiogenesis capabilities. Following that, the authors exploited surface dual crosslinking to simultaneously target ciliary body tissue (through a non-xanthine adenosine receptor antagonist (ZM241385)) and increase corneal epithelium penetration (by chitosan). The authors studied the anti-inflammatory properties of nanoparticles by measuring IL6 and MCP-1 levels and showed that by increasing the chitosan coating amount on the surface of ceria NPs, IL6 and monocyte chemoattractant protein-1 (MCP-1) production slightly increased. The authors showed that materials with dual functions, such as chitosan and ZM, may serve as antioxidants and anti-inflammatory agents, reducing inflammatory cytokines such as IL6 and MCP-1, when applied to the surface of ceria NPs. However, it should be noted that these metallic materials have the potential to form reactive oxygen species (ROS), which can sometimes lead to cytotoxicity in the eye (e.g., ZnO and Fe nanoparticles) (Zhu et al., 2019).

##### 3.2.1.3 Shape

The shape of the material and the way it affects how nanomaterials interact with innate immune cells is another crucial factor. There is no assurance that the innate immune system will not react negatively to the nanoparticles. Nanoparticle size and shape have the potential to stimulate macrophages to phagocytose and to consume and frustrate them, causing them to secrete an increasing amount of pro-inflammatory cytokines, eventually causing chronic inflammation, or even to stimulate DCs to mature and activate T cells (Bartneck et al., 2010; Niikura et al., 2013; Andorko and Jewell, 2017). According to Bartneck et al., macrophage absorption of gold nanorods is higher than that of nanospheres *via* the macropinocytosis process; nanorods elicited a stronger inflammatory response *in vivo* even after surface modification with polyethylene oxide (PEO) (Bartneck et al., 2010; Andorko and Jewell, 2017). Another intriguing work by Niikura et al. (2013) reported that the size and shape of gold nanoparticles (AuNPs) *in vitro* and *in vivo* may be used to tailor the responses of innate immune cells such as macrophages and DCs. Cells exposed to rod-shaped nanoparticles released cytokines *via* pro-inflammatory inflammasome pathways, such as IL-1 β and IL18. Cells treated with cubical and spherical nanoparticles produced TNF- α , IL6, and IL12 through various cytokine pathways (Niikura et al., 2013). The stiffness and aspect ratio of the nanoparticle increase as the shape of the particle changes from spherical to rod-shaped, cubical, hexagonal, or any other shape, and, as a result, macrophages trigging and the inflammatory response also increase based on the potential membrane damage by stiff particles in phagocytic cells (Woźniak et al., 2017; Li et al., 2021). In addition, it has been demonstrated in ocular studies that nanoparticles with a larger aspect ratio, such as those in the shape of fibers, carbon nanotubes, or 2D nanomaterial sharp sheets, are cytotoxic to eye tissue (Zhu et al., 2019).

##### 3.2.1.4 Surface charge

Another crucial factor is surface charge, which is particularly important for nanoparticles due to concerns regarding delivery, corona protein aggregation, and stability. Researchers have discovered that nanoparticles with a charge lower than −30 mV may have an anti-inflammatory impact on the immune system. A higher amount of negatively charged particles causes a lower immunogenic response and they may be immunosuppressive (Andorko and Jewell, 2017; Li H. et al., 2022; Wen et al., 2016; Getts et al., 2014). Despite the fact that positively charged particles may be more easily taken up by cells than negatively charged particles, they will indeed be more cytotoxic and trigger an inflammatory response in the cells (Zhu et al., 2019). Similarly, our group noted that surface charge plays an important role in the biodistribution of nanoparticles, while simultaneously triggering an immunogenic response (Huang and Chau, 2019a).

Yavuaz et al. used −50 mV negatively charged dexamethasone-PAMAM dendrimer conjugates for various ocular diseases affecting retinae such as AMD, DR, and glaucoma. The authors demonstrated that these dendrimers can effectively permeate to the back of the eye and release anti-inflammatory drugs in a sustained manner; however, the clearance time was quick. The authors suggested that the use of cationic dendrimers to determine the impact of surface charge on clearance time be taken into account (Yavuz et al., 2016). Additional information on the immunological characteristics of various engineered nanomaterials is discussed in the review of Dobrovolskaia (Dobrovolskaia and McNeil, 2007). Generation 3.5 PAMAM conjugated to glucosamine-negative dendrimers is one example that can inhibit human macrophages and DCs’ proinflammatory response and is immunosuppressive (Shaunak et al., 2004).

Lipid-based nanomaterials are another category of nanoparticles that have been widely used for ocular treatment, based on their controllable surface charge, especially as non-viral gene delivery systems. Due to their tendency to disassemble and aggregate, these materials are less stable in the eye compared to polymeric nanoparticles (Huang and Chau, 2019a). Lipid-based nanoparticles (LNPs) encapsulated with the model drug small interfering RNA (siRNA) were studied by our group to determine the impact of charge on intraocular distribution. We discovered that LNPs with neutral or negative charges exhibit problems with rapid clearance, whereas LNPs with a positive charge of 35 mV can diffuse through the retina and deliver the siRNA (Huang and Chau, 2019a; Huang and Chau, 2019b; Huang and Chau, 2021). In a different study conducted by our group, we found that siRNA-encapsulated LNPs with a positive surface charge of +33 mV did not cause activation of microglia cells or an inflammatory response (Xiaonan, 2020).

The particle clearance time and immunogenic reactions should be taken into account in ocular studies. The circulation and retention period of nanoparticles inside the eye can be prolonged by adopting a stealth technique that coats the nanoparticles with a hydrophilic substance called PEG. One example of this use is the systemic administration of PEG-stealth-PLA nanoparticles encapsulated with betamethasone for targeting the inflamed uvea and retina in a rat with EAU. The authors demonstrated that these stealth NPs reduced inflammation by the first day after administration, and their effects remained for 2 weeks. This period was significantly greater than their earlier experiment, which employed betamethasone/PLA NPs alone, which presented quick systemic phagocytosis clearance (Sakai et al., 2006; Sakai et al., 2011).

In order to inhibit the TLR-mediated innate immune pathway, Casey et al. (2019) used cargo-free nanoparticles of PLA and PLGA with different coatings of poly (ethylene-alt-maleic anhydride) (PEMA) and poly (vinyl alcohol) (PVA), different molecular weights, and different charges. The authors demonstrated that the PEMA coating caused a larger immunosuppression effect compared with the PVA coating. (Figure 4). The downregulation of pro-inflammatory cytokines and the suppression of TLR-mediated inflammation were accomplished by altering the physicochemical characteristics of the nanomaterials, such as the charge, molecular weight, and polymer composition.

#### 3.2.2 Immunomodulatory cargoes for nanoparticle

Using nanoparticles is the most common method for treating challenging ocular disorders related to the posterior part of the eye. By penetrating beyond ocular barriers, they can reach and distribute in the posterior segment. Based on the importance of chronic inflammatory diseases affecting the posterior region of the eye (e.g., glaucoma, AMD, and DR), the most common immunomodulatory cargoes used in nanoparticles are discussed in the following section. In light of this information, more efficient treatment strategies for chronic inflammatory eye disorders may be suggested, taking into account these potential cargoes and understanding the criteria for designing immunomodulatory nanoparticles.

##### 3.2.2.1 Steroids

The most common immunosuppressive agents are steroids. They have been used in a large number of commercial anti-inflammatory ocular drugs such as Bromfenac and INVELTYS™, both of which contain cross-linked PAA or chitosan polymer; they are widely used for treating post-ocular surgery inflammation, reducing post-surgery pain, and DED (Bowman et al., 2009; Schopf et al., 2015; Trattler and Hosseini, 2017; Kim et al., 2018; Gupta and Venkateswaran, 2021). Steroidal immunosuppressants have several drawbacks; they require high administration dosages, frequent injections in chronic ocular inflammatory diseases that lead to defects in the innate immune system, and other long-term side effects such as cataracts and IOP in the eye. By using immunomodulatory biomaterials as carriers instead of traditional inert carriers and alternative immunosuppressive drugs instead of steroids, such as anti-inflammatory cytokines, anti-inflammatory peptides, and nucleic acids, it is possible to overcome these problems in the eye.

##### 3.2.2.2 Biomolecules (proteins and peptides)

Another type of cargo used in ocular immunomodulatory treatment includes biomolecules such as proteins and peptides. However, there are drawbacks to employing these cargoes, for example, cytokine’s short half-life and biomolecules’ poor ocular bioavailability. Different immunosuppressive biomolecules such as TGF- β , α -melanocyte-stimulating hormone, and VIP are produced in the eye to retain its homeostasis and immune-privilege mechanism (Lajavardi et al., 2007). Each of these biomolecules, in an inflammatory state, may act as a possible immunosuppressant payload, restoring homeostasis. Several studies have used VIP as an immunosuppressant for the treatment of ocular inflammatory diseases (Lajavardi et al., 2007; Taylor, 2007). In uveitis, VIP-encapsulated liposome nanoparticles regulate macrophages and dendritic cells immune responses (Camelo et al., 2009). By combining VIP with liposome nanoparticles, the slow release of VIP effectively reduced the degree of uveitis and pro-inflammatory cytokines and prevented retinal damage (Camelo et al., 2009). Another immunomodulatory peptide from fungi, cyclosporine A (Cy A), is being investigated as an immunosuppressant for inflammatory ocular conditions because it suppresses the proinflammatory cytokine IL2 (Nussenblatt and Palestine, 1986; Serda et al., 1990; Gupta and Sahu, 2001; Rumelt et al., 2002; Lallemand et al., 2003; Yenice et al., 2008). A study by Yenice et al. (2008) investigated the bioavailability of Cy A in the cornea with regard to HA-coated PCL nanoparticles. In this respect, regulatory cytokines, and their prolonged release in the site of inflammation, may treat ocular inflammatory diseases by polarizing innate immune cells in an anti-inflammatory manner. IL4, IL13, and IL10 are the most frequently mentioned anti-inflammatory cytokines that are used as immunomodulatory payloads for the innate immune response and M2 polarization (Reeves et al., 2015; Schirmer et al., 2016; Cha et al., 2017; Riabov et al., 2017; He et al., 2019; Li et al., 2021).

As previously mentioned, TNF- α is a pro-inflammatory cytokine that is produced in large amounts in inflammatory conditions; thus, blocking its receptor may be a promising way to suppress inflammation. For instance, Woo Ji et al. developed a TNF- α blocker HL036 peptide for the topical treatment of dry eye, and showed that it could effectively eliminate TNF- α on the ocular surface and suppress inflammation by lowering the IFN- γ , IL6, and IL21 proinflammatory cytokines (Ji et al., 2013).

A recent study was conducted on the dynamics of microglia polarization and their regulation by an anti-inflammatory mixture of cytokines IL4/IL13 or the antitumor agent bicyclic nojirimycin derivative (1R)-1-dodecylsulfinyl-5N,6O-oxomethylidenenojirimycin (R-DS-ONJ) in a mouse DR model. The authors reported that using M2 cytokines or R-DS-ONJ as a modulating agent in the environment of activated microglia cells reduced retinal degeneration and inflammatory progression both *in vitro* and *in vivo* (Arroba et al., 1862). Further research can be conducted in this area to determine if inflammatory microglia targeting can be used as a therapeutic method to postpone or stop the impairment of visual function in DR.

##### 3.2.2.3 Nucleic acids

Gene therapy for ocular inflammation may be an excellent option, even in clinical trials, and may use DNA, RNA, aptamers, and oligonucleotides. The stability of nucleic acid and the efficiency of its targeted delivery to minimize off-target effects are two challenges in this approach (Guzman-Aranguez et al., 2013; Raghunath and Perumal, 2015).

Viral and non-viral vectors are the two main types of nucleic acid delivery methods. Although viral vectors have been widely used as nucleic acid delivery methods, their immunogenicity, which may result in inflammation by inducing an immune response, is a major concern when employing them in clinical studies (Bordet and Behar-Cohen, 2019). These viruses include adenoviruses (AVs), adeno-associated viruses (AAVs), and lentiviruses. AAVs have been used in several studies on ocular gene therapy and chronic ocular disorders, although it has been demonstrated that they produce a mild immune response (Timmers et al., 2020). The first FDA-approved ocular gene therapy product, Luxturna®, also used an AAV2 vector and targeted diseases caused by the mutation of RPE65 genes such as retinitis pigmentosa (Russell et al., 2017). In this way, the type of vector, route of administration, and viral dosage cause different immune responses. For instance, the intravitreal route showed a higher immune response in comparison to other routes, and subretinal injection showed a lower immune response (Wasnik and Thool, 2022). An interesting study on the immunomodulation of uveitis by gene therapy is the use of a mutant serotype 8 adeno-associated virus (AAV8) (Y733F)-chicken β-actin (CBA)-MIF vector to express the macrophage migration inhibitory factor (MIF), which is an important cytokine for regulating macrophage function and T cell activation (Yang et al., 2016). It has been shown that MIF is critical for modulating ocular inflammation in EAU, linked to the Notch signaling pathway; this may be a promising therapeutic approach for uveitis.

Recently, Gilger and Hirsch (2022) reviewed the potential of using the human leukocyte antigen G (HLA-G) gene in AAV vectors for ocular inflammatory treatments. HLA-G is an important anti-inflammatory agent that can suppress the inflammatory function of innate immune cells such as neutrophils, dendritic cells, macrophages, and natural killer cells. The authors stated that, based on the anti-vascular performance of the HLA-G, it could be a great target for the treatment of different ocular diseases including corneal inflammation, corneal graft rejection, DED, uveitis, AMD, and DR (Gilger and Hirsch, 2022).

Nevertheless, based on concerns regarding the immunogenicity of viral vectors (Bordet and Behar-Cohen, 2019), non-viral vectors (with low immunogenic risk) are alternatives for gene therapy, especially in immunomodulatory approaches. LNPs, nano polymers, and self-assembly peptides with nucleic acid are the most common non-viral delivery systems in this area. There are several studies and clinical trials on ocular gene therapy for immunomodulatory approaches using siRNA, miRNA, mRNA, DNA, and plasmid DNA (Ghoraba et al., 2022).

The use of siRNA in different ocular inflammatory diseases was reported for silencing different innate immune cell-mediated inflammatory genes (Guzman-Aranguez et al., 2013). For instance, SYL040012 (bamosiran) is a commercial siRNA topical drug used to suppress the β-Adrenergic Receptor 2 as well as to lower intraocular pressure in glaucoma (Pintor, 2012; Bordet and Behar-Cohen, 2019; Ghoraba et al., 2022). For treating neovascularization-related disorders such as retinopathy and AMD, an important key regulatory agent is VEGF. Inhibition of VEGF was achieved by using recombinant AAV-mediated soluble VEGF receptor 1 (sFlt-1) expression (Bainbridge et al., 2002; Rota et al., 2004; Shen et al., 2006) or siRNA-targeting VEGF (Reich et al., 2003; Jiang et al., 2009; Ashikari et al., 2010). It was shown that neovascularization was reduced in the retina and choroidal, respectively. Interestingly, Ryoo et al. (2017) developed a novel siRNA-based anti-VEGF nanoball that was composed of siRNA-targeting VEGF as the core hydrogel with a coating of branched PEI and HA, achieved by applying an electrical force, for the treatment of choroidal neovascularization in AMD. The authors demonstrated excellent therapeutic results over 2 weeks as a result of intravitreal injection and targeting efficiency in the sub-retinal space *via* CD44 receptor endocytosis. AAV-mediated gene delivery of pigment epithelium derived factor (PEDF) reduced neovascularization in the choroid (Mori et al., 2002). The potential role of RTP801 in retinopathy was also studied, and the results indicated that by inhibition of its expression, the development of retinopathy was significantly reduced (Brafman et al., 2004).

miRNA is also used for regulating inflammatory-mediated genes in different diseases in the eye (Raghunath and Perumal, 2015). For instance, in AMD treatment, it has been shown that the upregulation of miR-9, miR-125b, miR-146a, and miR-155, alone or in combination, regulates the innate immune system and inflammation signals *via* the NF- κ B or other pathogen pathways (Li et al., 2011; Lukiw et al., 2012; Kutty et al., 2013; Raghunath and Perumal, 2015; Pogue and Lukiw, 2018). In an interesting work by Zou et al., the impact of miRNA on uveitis treatment was studied. The authors demonstrated that transfecting the DCs using miR-155 inhibits the expression of pro-inflammatory cytokines such as IL6 and IL-1 β , and increases the production of the anti-inflammatory IL10 cytokine (Zhou et al., 2012). Park et al. (2009) showed that using the expression plasmid of plasminogen Kringle 5 (K5) encapsulated in PLGA/chitosan nanoparticles has the potential to treat retinal inflammation in DR by downregulating retinal VEGF expression and the anti-inflammatory effect (Diebold and Calonge, 2010). Our group’s review study and research on effective siRNA delivery systems for ocular therapy provides more information regarding nucleic acid ocular delivery cargoes (Huang and Chau, 2019a; Huang and Chau, 2019b; Huang and Chau, 2021).

Messenger RNA (mRNA) is another type of nucleic acid that has been widely used in gene therapy. It allows faster protein translation as it does not require trafficking of the cargo to the cell nucleus in comparison to DNA. A primary anti-inflammatory cytokine is IL10, and delivery of the IL10 gene to the disorders causing ocular inflammation may be an effective therapy. To treat ocular inflammation, one method employed is to distribute IL10 mRNA using solid lipid nanoparticles (SLN) and various polymeric ligands such as dextran or HA for long-term stability (Gómez-Aguado et al., 2021). In this study, the authors used mRNA and plasmid DNA (pDNA) for *de novo* production of IL10 and showed that the mRNA systems induced a higher expression of IL10 in corneal epithelial cells than pDNA systems, and that they suppress corneal inflammation.

### 3.3 Hybrid systems of biomaterial carriers and different cargoes

New drug delivery systems that combine several drug delivery techniques such as hydrogel and nano systems, have shown intriguing results in recent years, leading to the development of more intelligent and controlled drug delivery systems. In ophthalmic drug delivery systems, it is important to reduce side effects and increase treatment efficiency and bioavailability by minimal injection or surgery in the eye (Sharma et al., 2016). One example of these hybrid scaffolds is provided by du Toit et al. (2013). The hybrid system employed was an indomethacin anti-inflammatory non-steroidal drug encapsulated in a lipoidal-chitosan-PCL nano system with a size of 140.7 nm and a positive surface charge for targeting the inflammatory disorder in the posterior part of the eye. The authors demonstrated that, in comparison to the chitosan-PCL nano system alone, the composite lipoidal nano system suppressed the inflammation by decreasing the amount of NF- κ B and by enhancing the inflammatory cellular uptake of the drug. Gomes do Santos et al. also developed a hybrid system comprising antisense TGF- β 2 phosphorothioate oligonucleotides/PEI nanoparticle that was encapsulated in PLGA microspheres, namely, “Trojan” microspheres. The authors stated that this “Trojan” system could prevent post-surgery fibrosis for 42 days (Gomes dos Santos et al., 2006). The DNA/PLGA hybrid hydrogel (HDNA), which is used to sustain the release of water-insoluble anti-inflammatory drugs such as dexamethasone, is another example of these systems. In animal models of allergic conjunctivitis, once-daily topical treatment using this porous hybrid structure outperformed multiple treatments using commercial dexamethasone drops (Ren et al., 2019).

## 4 Conclusion

In ocular inflammatory illnesses, innate immune cells play a key role in removing the inflammatory pathogen or infection and subsequent regeneration of the inflamed region by homeostatic action and fibrosis development. Since the eye is extremely sensitive to inflammation and injury, it is critical to maintain its homeostasis and to avoid any chronic or harmful inflammation that can lead to ocular disorders such as DED, uveitis, glaucoma, AMD, as well as any other chronic inflammation that can result in eye tissue degeneration and blindness. Further research regarding the eye’s immunological response is required for designing tolerogenic biomaterials that act as intraocular lenses, cellular scaffolds, therapeutic molecule depots, or carriers of gene therapies. The discussion presented in this article sheds light on the potential use of biomaterials to direct immune responses toward favorable treatment outcomes.

Biomaterials can be utilized as manipulating agents for interacting with ocular innate immune cells and directing pro- or anti-inflammatory responses by using them either cargo-less or cargo-loaded. In this review, we discussed several physiochemical features of biomaterials such as mechanical strength, size, shape, charge, wettability, and biodegradability, as well as how each of these factors might program the fate of the innate immune cells’ phase by inhibiting or inducing special receptors and signaling pathways. These physiochemical parameters also affect the biodistribution and the interaction between the target cells and the cargoes. While previous efforts have focused on the intended action of the carrier, the influence of these parameters on the immune response has been neglected. They are often “discovered” as a surprise or treated as an unexpected side effect.

However, one of the most difficult issues to address is determining how to identify the influence of each physiochemical feature on the underlying biomaterial-related immune response and what cross-talks exist between them. More study and deeper understanding are required to determine the precise cell-biomaterial interaction mechanism by combining these physiochemical features and determining the effect of combining them on innate immune system programming. In the future, machine learning and artificial intelligence (AI) will be useful to forecast the ocular innate immune system’s reaction to a given biomaterial with defined features. A combination of gene therapies and immunomodulatory biomaterials carriers could be also a promising method for programming targeted overacting pro-inflammatory innate immune cells in a downregulating manner to retain their anti-inflammatory action, without side-effects on other immune cells’ fighting action.

Overall, there is significant therapeutic potential in ocular inflammatory diseases by focusing on triggering the anti-inflammatory phase of ocular-activated innate immune cells and providing homeostasis and tissue regeneration signals to the eye *via* immunomodulatory biomaterials that can sustain the release of novel therapeutic agents such as nucleic acids, biomolecules, or MSCs for an extended period of time with only one administration.

## Glossary

NTs: Neutrophils
MG: Microglia
Mφs: Macrophages
DCs: Dendritic Cells
cDC: conventional DC
pDC: plasmacytoid DC
MSCs: Mesenchymal stem cells
BM-MSCs: Bone marrow-derived MSCs
LCs: Langerhans cells
AM-MSCs: Adipose tissue-derived MSCs
LSCs: Limbal epithelial stem cells
APCs: Antigen-presenting cells
ECM: Extracellular matrix
AMD: Age-related macular degeneration
DED: Dry eye disease
DR: Diabetic retinopathy
EAU: Experimental autoimmune uveitis
OHT: Ocular hypertension
IOP: Intraocular pressure
RP: Retinitis pigmentosa
RGCs: Retinal ganglion cells
MGD: Meibomian gland dysfunction
DAMP: Danger-associated molecular pattern
PAMP: Pathogen-associated molecular pattern
NETs: Neutrophil extracellular traps
ACAID: Anterior chamber-associated immune deviation
ADP/ATP: Adenosine di/tri-phosphate
NF-κB: Nuclear factor kappa B
IOL: Intraocular lens
FA: Fluocinolone acetonide
FBR: Foreign body response
HLA-G: Human leukocyte antigen G
HMGB1: High mobility group protein B1
LPS: Lipopolysaccharides
IDO: Indoleamine 2, 3-dioxygenase
PEDF: Pigment epithelium derived factor
PRR: Pattern-recognition receptors
LAIR-1: Leukocyte-associated immunoglobulin-like receptor-1
RANTES: Regulated upon activation, normal T cell expressed and secreted
Ly6C: Lymphocyte antigen 6 complex
OVA: Ovalbumin
HA: Hyaluronate
GelMA: Methacrylate-gelatin
AVs: Adenoviruses
AAVs: Adeno-associated viruses
mRNA: Messenger RNA
miRNA: Micro RNA
siRNA: small interfering RNA
pDNA: Plasmid DNA
NPs: Nanoparticles
MPs: Microparticles
LNPs: Lipid-based nanoparticles
SLN: Solid lipid nanoparticles
AuNPs: Gold nanoparticle
AI: Artificial intelligence
AM: Amniotic membrane
AIM2: Absent in melanoma 2
TNF-⍺: Tumor necrosis factor-alpha
IFN-γ: Interferon gamma
TGF-β: Transforming growth factor-β
iNOS: inducible nitric oxide synthase
IL-: Interleukin-
P2Y12: P2 purinergic receptor
Caspase-1: Cysteine-aspartic acid protease-1
Iba-1: Ionized calcium-binding adapter molecule 1
APTES: Aminopropyltriethoxylsilane
ARG: Arginase
ILM: Internal limiting membrane
MCP-1: Monocyte chemoattractant protein-1
IgG: Immunoglobulin G
CCL-: C-C motif chemokine ligand
CCR-: C-C chemokine receptor
CD-: Cluster of differentiation
CD200R: Cluster of differentiation-200 receptor
MHCII: Major histocompatibility complex class II
CNTF: Ciliary neurotrophic factor
CNV: Choroidal neovascularization
COX-: Cyclooxygenase
CR2-fH: Complement receptor 2 and factor H
CXCL-: C-X-C motif chemokine ligand
Cy A: Cyclosporine A
PD-L1: Programmed cell death-ligand 1
NLR: Nod-like receptors
VEGF: Vascular endothelial growth factor
TLR-: Toll-like receptor
ROS: Reactive oxygen species
TRPV: Transient receptor potential vanilloid
K 5: Kringle 5
VIP: Vasoactive intestinal peptide
LBL: Layer-by-layer
Siglec-H: Sialic acid binding immunoglobulin-like lectin H
MIF: Migration inhibitory factor
MMP: Matrix metalloproteinase
MyD88: Myeloid differentiation primary response 88
PCO: Posterior capsular opacification
RGD: Arginyl-glycyl-aspartic acid
PAA: Polyacrylic acid
PAMAM: Poly (amidoamine)
mPEG-PLGA-BOX: Methoxy-poly (ethylene glycol)-block-poly (lactic-co-glycolic acid)
PBT: Polybutylene terephthalate
PCL: Poly-caprolactone
PEG: Polyethylene glycol
PEI: Polyethylenimine
PEMA: Poly (ethylene-alt-maleic anhydride)
PEO: Polyethylene oxide
PHEMA: Poly (2-hydroxyethyl methacrylate)
PLA: Polylactic acid
PLGA: Poly(lactide-co-glycolide)
PMMA: Poly (methyl methacrylate)
PVA: Poly (vinyl alcohol)

## References

[B1] Abu-Ain M. S. Webber S. K. (2010). The biological bandage contact lens: A novel technique for using the amniotic membrane in the treatment of persistent corneal epithelial defects. Eye 24 (7), 1306–1307. 10.1038/eye.2010.1 20111063

[B2] Adamson P. Wilde T. Dobrzynski E. Sychterz C. Polsky R. Kurali E. (2016). Single ocular injection of a sustained-release anti-VEGF delivers 6months pharmacokinetics and efficacy in a primate laser CNV model. J. Control Release 244, 1–13. 10.1016/j.jconrel.2016.10.026 27810558 PMC5494198

[B3] Ahn H. Kim J. Jeung E. B. Lee G. S. (2014). Dimethyl sulfoxide inhibits NLRP3 inflammasome activation. Immunobiology 219 (4), 315–322. 10.1016/j.imbio.2013.11.003 24380723

[B4] Allyn M. M. Luo R. H. Hellwarth E. B. Swindle-Reilly K. E. (2022). Considerations for polymers used in ocular drug delivery. Front. Med. (Lausanne) 8, 787644. 10.3389/fmed.2021.787644 35155469 PMC8831705

[B5] Anderson J. M. (2003). Biological responses to materials. Annu. Rev. Mater. Res. 31, 81–110. 10.1146/annurev.matsci.31.1.81

[B6] Anderson J. M. Rodriguez A. Chang D. T. (2008). Foreign body reaction to biomaterials. Semin. Immunol. 20 (2), 86–100. 10.1016/j.smim.2007.11.004 18162407 PMC2327202

[B7] Andorko J. I. Jewell C. M. (2017). Designing biomaterials with immunomodulatory properties for tissue engineering and regenerative medicine. Bioeng. Transl. Med. 2 (2), 139–155. 10.1002/btm2.10063 28932817 PMC5579731

[B8] Andrés-Guerrero V. Zong M. Ramsay E. Rojas B. Sarkhel S. Gallego B. (2015). Novel biodegradable polyesteramide microspheres for controlled drug delivery in Ophthalmology. J. Control. Release 211, 105–117. 10.1016/j.jconrel.2015.05.279 26003040

[B9] Arroba A. I. Alcalde-Estevez E. García-Ramírez M. Cazzoni D. de la Villa P. Sánchez-Fernández E. M. (1862). Modulation of microglia polarization dynamics during diabetic retinopathy in db/db mice. Biochimica Biophysica Acta (BBA) - Mol. Basis Dis. 1862 (9), 1663–1674. 10.1016/j.bbadis.2016.05.024 27267343

[B10] Ashikari M. Tokoro M. Itaya M. Nozaki M. Ogura Y. (2010). Suppression of laser-induced choroidal neovascularization by nontargeted siRNA. Invest. Ophthalmol. Vis. Sci. 51 (7), 3820–3824. 10.1167/iovs.09-5121 20130283

[B11] Aşık M. D. Doğan Aşık M. Uğurlu N. Yülek F. Tuncer S. Türk M. (2013). Ketorolac tromethamine loaded chitosan nanoparticles as a nanotherapeutic system for ocular diseases. Hacettepe J. Biol. Chem. 41 (1), 81–86.

[B12] Bainbridge J. W. B. Mistry A. de Alwis M. Paleolog E. Baker A. Thrasher A. J. (2002). Inhibition of retinal neovascularisation by gene transfer of soluble VEGF receptor sFlt-1. Gene Ther. 9 (5), 320–326. 10.1038/sj.gt.3301680 11938451

[B13] Bartneck M. Keul H. A. Singh S. Czaja K. Bornemann J. Bockstaller M. (2010). Rapid uptake of gold nanorods by primary human blood phagocytes and immunomodulatory effects of surface chemistry. ACS Nano 4 (6), 3073–3086. 10.1021/nn100262h 20507158

[B14] Bauernfeind F. Rieger A. Schildberg F. A. Knolle P. A. Schmid-Burgk J. L. Hornung V. (2012). NLRP3 inflammasome activity is negatively controlled by miR-223. J. Immunol. 189 (8), 4175–4181. 10.4049/jimmunol.1201516 22984082

[B15] Beeken L. J. Ting D. S. J. Sidney L. E. (2021). Potential of mesenchymal stem cells as topical immunomodulatory cell therapies for ocular surface inflammatory disorders. Stem Cells Transl. Med. 10 (1), 39–49. 10.1002/sctm.20-0118 32896982 PMC7780815

[B16] Belhaj M. Annamalai B. Parsons N. Shuler A. Potts J. Rohrer B. (2020). Encapsulated cell technology for the delivery of biologics to the mouse eye. J. Vis. Exp. 2020 (157). 10.3791/60162 32281978

[B17] Blume Z. I. Lambert J. M. Lovel A. G. Mitchell D. M. (2020). Microglia in the developing retina couple phagocytosis with the progression of apoptosis via P2RY12 signaling. Dev. Dyn. 249 (6), 723–740. 10.1002/dvdy.163 32072708 PMC8714022

[B18] Bordet T. Behar-Cohen F. (2019). Ocular gene therapies in clinical practice: Viral vectors and nonviral alternatives. Drug Discov. Today 24 (8), 1685–1693. 10.1016/j.drudis.2019.05.038 31173914

[B19] Bowman L. M. Si E. Pang J. Archibald R. Friedlaender M. (2009). Development of a topical polymeric mucoadhesive ocular delivery system for azithromycin. J. Ocul. Pharmacol. Ther. 25 (2), 133–139. 10.1089/jop.2008.0066 19284320

[B20] Brafman A. Mett I. Shafir M. Gottlieb H. Damari G. Gozlan-Kelner S. (2004). Inhibition of oxygen-induced retinopathy in RTP801-deficient mice. Invest. Ophthalmol. Vis. Sci. 45 (10), 3796–3805. 10.1167/iovs.04-0052 15452091

[B21] Camelo S. Lajavardi L. Bochot A. Goldenberg B. Naud M. C. Brunel N. (2009). Protective effect of intravitreal injection of vasoactive intestinal peptide-loaded liposomes on experimental autoimmune uveoretinitis. J. Ocul. Pharmacol. Ther. 25 (1), 9–21. 10.1089/jop.2008.0074 19232006

[B22] Carrasquillo K. G. Ricker J. A. Rigas I. K. Miller J. W. Gragoudas E. S. Adamis A. P. (2003). Controlled delivery of the anti-VEGF aptamer EYE001 with poly(lactic-co-glycolic)acid microspheres. Invest. Ophthalmol. Vis. Sci. 44 (1), 290–299. 10.1167/iovs.01-1156 12506087

[B23] Casey L. M. Kakade S. Decker J. T. Rose J. A. Deans K. Shea L. D. (2019). Cargo-less nanoparticles program innate immune cell responses to toll-like receptor activation. Biomaterials 218, 119333. 10.1016/j.biomaterials.2019.119333 31301576 PMC6679939

[B24] Casey-Power S. Ryan R. Behl G. McLoughlin P. Byrne M. E. Fitzhenry L. (2022). Hyaluronic acid: Its versatile use in ocular drug delivery with a specific focus on hyaluronic acid-based polyelectrolyte complexes. Pharmaceutics 14 (7), 1479. 10.3390/pharmaceutics14071479 35890371 PMC9323903

[B25] Cejka C. Holan V. Trosan P. Zajicova A. Javorkova E. Cejkova J. (2016). The favorable effect of mesenchymal stem cell treatment on the antioxidant protective mechanism in the corneal epithelium and renewal of corneal optical properties changed after alkali burns. Oxid. Med. Cell Longev. 2016, 5843809. 10.1155/2016/5843809 27057279 PMC4736412

[B26] Cha B. H. Shin S. R. Leijten J. Li Y. C. Singh S. Liu J. C. (2017). Integrin-mediated interactions control macrophage polarization in 3D hydrogels. Adv. Healthc. Mater 6 (21), 1700289. 10.1002/adhm.201700289 PMC5677560 28782184

[B27] Chen B. M. Cheng T. L. Roffler S. R. (2021). Polyethylene glycol immunogenicity: Theoretical, clinical, and practical aspects of anti-polyethylene glycol antibodies. ACS Nano 15, 14022–14048. 10.1021/acsnano.1c05922 34469112

[B28] Chen M. Luo C. Zhao J. Devarajan G. Xu H. (2019). Immune regulation in the aging retina. Prog. Retin Eye Res. 69, 159–172. 10.1016/j.preteyeres.2018.10.003 30352305 PMC6373845

[B29] Chew E. Y. Clemons T. E. Peto T. Sallo F. B. Ingerman A. Tao W. (2015). Ciliary neurotrophic factor for macular telangiectasia type 2: Results from a phase 1 safety trial. Am. J. Ophthalmol. 159 (4), 659–666.e1. 10.1016/j.ajo.2014.12.013 25528956 PMC4361328

[B30] Chinnery H. R. Mcmenamin P. G. Dando S. J. (1947). Macrophage physiology in the eye. Pflugers Arch. 469 (3-4), 501–515. 10.1007/s00424-017-1947-5 28233124

[B31] Chittasupho C. Posritong P. Ariyawong P. (2019). Stability, cytotoxicity, and retinal pigment epithelial cell binding of hyaluronic acid-coated PLGA nanoparticles encapsulating lutein. AAPS PharmSciTech 20 (1), 4–13. 10.1208/s12249-018-1256-0 30560323

[B32] Cohen S. Yoshioka T. Lucarelli M. Hwang L. H. Langer R. (1991). Controlled delivery systems for proteins based on poly(lactic/glycolic acid) microspheres. Pharm. Res. 8 (6), 713–720. 10.1023/a:1015841715384 2062800

[B33] Cuartero M. I. Ballesteros I. Moraga A. Nombela F. Vivancos J. Hamilton J. A. (2013). N2 neutrophils, novel players in brain inflammation after stroke: Modulation by the pparγ agonist rosiglitazone. Stroke 44 (12), 3498–3508. 10.1161/STROKEAHA.113.002470 24135932

[B34] Cui L. Yao Y. Yim E. K. F. (2021). The effects of surface topography modification on hydrogel properties. Apl. Bioeng. 5 (3), 031509. 10.1063/5.0046076 34368603 PMC8318605

[B35] Dick A. D. Carter D. Robertson M. Broderick C. Hughes E. Forrester J. v. (2003). Control of myeloid activity during retinal inflammation. J. Leukoc. Biol. 74 (2), 161–166. 10.1189/jlb.1102535 12885931

[B36] Das S. Ahmad Z. Suryawanshi A. Kumar A. (2022). Innate immunity dysregulation in aging eye and therapeutic interventions. Ageing Res. Rev. 82, 101768. 10.1016/j.arr.2022.101768 36280210

[B37] Davis M. E. Brewster M. E. (2004). Cyclodextrin-based pharmaceutics: Past, present and future. Nat. Rev. Drug Discov. 3 (12), 1023–1035. 10.1038/nrd1576 15573101

[B38] de La Fuente M. Seijo B. Alonso M. J. (2008). Novel hyaluronic acid-chitosan nanoparticles for ocular gene therapy. Invest. Ophthalmol. Vis. Sci. 49 (5), 2016–2024. 10.1167/iovs.07-1077 18436835

[B39] Dempsey P. W. Vaidya S. A. Cheng G. (2003). The Art of War: Innate and adaptive immune responses. Cell. Mol. Life Sci. CMLS 1260 (12), 2604–2621. 10.1007/s00018-003-3180-y PMC11138847 14685686

[B40] Diebold Y. Calonge M. (2010). Applications of nanoparticles in ophthalmology. Prog. Retin Eye Res. 29 (6), 596–609. 10.1016/j.preteyeres.2010.08.002 20826225

[B41] Dobrovolskaia M. A. McNeil S. E. (2007). Immunological properties of engineered nanomaterials. Nat. Nanotechnol. 2 (8), 469–478. 10.1038/nnano.2007.223 18654343

[B42] Domala A. Bale S. Godugu C. (2019). Protective effects of nanoceria in imiquimod induced psoriasis by inhibiting the inflammatory responses. Nanomedicine 15 (1), 5–22. 10.2217/nnm-2018-0515 31868114

[B43] Donaruma L. G. (1988). Definitions in biomaterials, D. F. Williams, Ed., Elsevier, Amsterdam, 1987, 72 pp. JPoSL 26, 414.

[B44] D’Orazio T. J. Mayhew E. Niederkorn J. Y. (2001). Ocular immune privilege promoted by the presentation of peptide on tolerogenic B cells in the spleen. II. Evidence for presentation by qa-1. J. Immunol. 166 (1), 26–32. 10.4049/jimmunol.166.1.26 11123273

[B45] D’orazio T. J. Niederkorn J. Y. (1998). The nature of antigen in the eye has a profound effect on the cytokine milieu and resultant immune response. Eur. J. Immunol. 28 (5), 1544–1553. 10.1002/(SICI)1521-4141(199805)28:05<1544::AID-IMMU1544>3.0.CO;2-P 9603459

[B46] Du H. Bartleson J. M. Butenko S. Alonso V. Liu W. F. Winer D. A. (2022). Tuning immunity through tissue mechanotransduction. Nat. Rev. Immunol., 1–15. 10.1038/s41577-022-00761-w 35974148 PMC9379893

[B47] du Toit L. C. Govender T. Carmichael T. Kumar P. Choonara Y. E. Pillay V. (2013). Design of an anti-inflammatory composite nanosystem and evaluation of its potential for ocular drug delivery. J. Pharm. Sci. 102 (8), 2780–2805. 10.1002/jps.23650 23828405

[B48] Eitan E. Hutchison E. Greig N. Tweedie D. Celik H. Ghosh S. (2015). Combination therapy with lenalidomide and nanoceria ameliorates CNS autoimmunity. Exp. Neurol. 273, 151–160. Elsevier. 10.1016/j.expneurol.2015.08.008 26277686 PMC4644463

[B49] Enríquez de Salamanca A. Diebold Y. Calonge M. García-Vazquez C. Callejo S. Vila A. (2006). Chitosan nanoparticles as a potential drug delivery system for the ocular surface: Toxicity, uptake mechanism and *in vivo* tolerance. science 47 (4), 1416–1425. 10.1167/iovs.05-0495 16565375

[B50] Estúa-Acosta G. A. Zamora-Ortiz R. Buentello-Volante B. García-Mejía M. Garfias Y. (2019). Neutrophil extracellular traps: Current perspectives in the eye. Cells 8 (9), 979. 10.3390/cells8090979 31461831 PMC6769795

[B51] Fan W. Huang W. Chen J. Li N. Mao L. Hou S. (2022). Retinal microglia: Functions and diseases. Immunology 166 (3), 268–286. 10.1111/imm.13479 35403700

[B52] Farid M. Sabeti S. Minckler D. S. (2020). Histopathological study of an explanted novel artificial corneal device. Cornea 39 (7), 915–918. 10.1097/ICO.0000000000002261 32040006

[B53] Fernández-Albarral J. A. Salazar J. J. Hoz R. Marco E. M. Martín-Sánchez B. Flores-Salguero E. (2021). Retinal molecular changes are associated with neuroinflammation and loss of RGCs in an experimental model of glaucoma. Int. J. Mol. Sci. 22 (4), 2066. 33669765 10.3390/ijms22042066 PMC7922243

[B54] Formisano N. van der Putten C. Grant R. Sahin G. Truckenmüller R. K. Bouten C. V. C. (2021). Mechanical properties of bioengineered corneal stroma. Adv. Healthc. Mater 10 (20), e2100972. 10.1002/adhm.202100972 34369098 PMC11468718

[B55] Ganugula R. Arora M. Lepiz M. A. Niu Y. Mallick B. K. Pflugfelder S. C. (2020). Systemic anti-inflammatory therapy aided by double-headed nanoparticles in a canine model of acute intraocular inflammation. Sci. Adv. 6 (35), eabb7878. 10.1126/sciadv.abb7878 32923645 PMC7449680

[B56] Gao J. Yu X. Wang X. He Y. Ding J. (2022). Biomaterial–related cell microenvironment in tissue engineering and regenerative medicine. Engineering 13, 31–45. 10.1016/j.eng.2021.11.025

[B57] García-Culebras A. Durán-Laforet V. Peña-Martínez C. Ballesteros I. Pradillo J. M. Díaz-Guzmán J. (2018). Myeloid cells as therapeutic targets in neuroinflammation after stroke: Specific roles of neutrophils and neutrophil–platelet interactions. J. Cereb. Blood Flow Metabolism 38 (12), 2150–2164. 10.1177/0271678X18795789 PMC6282223 30129391

[B58] Garzón H. Suárez L. J. Muñoz S. Cardona J. Fontalvo M. Alfonso-Rodríguez C. A. (2022). Biomaterials used for periodontal disease treatment: Focusing on immunomodulatory properties. Int. J. Biomater. 2022, 7693793. 10.1155/2022/7693793 35528847 PMC9072036

[B59] Gavini E. Chetoni P. Cossu M. Alvarez M. G. Saettone M. F. Giunchedi P. (2004). PLGA microspheres for the ocular delivery of a peptide drug, vancomycin using emulsification/spray-drying as the preparation method: *In vitro*/*in vivo* studies. Eur. J. Pharm. Biopharm. 57 (2), 207–212. 10.1016/j.ejpb.2003.10.018 15018976

[B60] Gebe J. A. Yadava K. Ruppert S. M. Marshall P. Hill P. Falk B. A. (2017). Modified high-molecular-weight hyaluronan promotes allergen-specific immune tolerance. Am. J. Respir. Cell Mol. Biol. 56 (1), 109–120. 10.1165/rcmb.2016-0111OC 27598620 PMC5248962

[B61] Getts D. R. Terry R. L. Getts M. T. Deffrasnes C. Müller M. Vreden C. (2014). Therapeutic inflammatory monocyte modulation using immune-modifying microparticles. Sci. Transl. Med. 6 (219), 219ra7. 10.1126/scitranslmed.3007563 PMC3973033 24431111

[B62] Ghasemi H. Ghazanfari T. Yaraee R. Owlia P. Hassan Z. M. Faghihzadeh S. (2012). Roles of IL-10 in ocular inflammations: A review. Ocul. Immunol. Inflamm. 20 (6), 406–418. 10.3109/09273948.2012.723109 23163602

[B63] Ghoraba H. H. Akhavanrezayat A. Karaca I. Yavari N. Lajevardi S. Hwang J. (2022). Ocular gene therapy: A literature review with special focus on immune and inflammatory responses. Clin. Ophthalmol. 16, 1753–1771. 10.2147/OPTH.S364200 35685379 PMC9173725

[B64] Ghosh S. Padmanabhan A. Vaidya T. Watson A. M. Bhutto I. A. Hose S. (2019). Neutrophils homing into the retina trigger pathology in early age-related macular degeneration. Commun. Biol. 2 (1), 348. 10.1038/s42003-019-0588-y 31552301 PMC6754381

[B65] Gilger B. C. Hirsch M. L. (2022). Therapeutic applications of adeno-associated virus (AAV) gene transfer of HLA-G in the eye. Int. J. Mol. Sci. 23 (7), 3465. 10.3390/ijms23073465 35408825 PMC8998501

[B66] Ginhoux F. Lim S. Hoeffel G. Low D. Huber T. (2013). Origin and differentiation of microglia. Front. Cell Neurosci. 7, 45. 10.3389/fncel.2013.00045 23616747 PMC3627983

[B67] Glybina I. v. Kennedy A. Ashton P. Abrams G. W. Iezzi R. (2010). Intravitreous delivery of the corticosteroid fluocinolone acetonide attenuates retinal degeneration in S334ter-4 rats. Invest. Ophthalmol. Vis. Sci. 51 (8), 4243–4252. 10.1167/iovs.09-4492 20220055 PMC2910647

[B68] Glybina I. v. Kennedy A. Ashton P. Abrams G. W. Iezzi R. (2009). Photoreceptor neuroprotection in RCS rats via low-dose intravitreal sustained-delivery of fluocinolone acetonide. Invest. Ophthalmol. Vis. Sci. 50 (10), 4847–4857. 10.1167/iovs.08-2831 19407016

[B69] Gomes dos Santos A. L. Bochot A. Doyle A. Tsapis N. Siepmann J. Siepmann F. (2006). Sustained release of nanosized complexes of polyethylenimine and anti-TGF-β2 oligonucleotide improves the outcome of glaucoma surgery. J. Control. Release 112 (3), 369–381. 10.1016/j.jconrel.2006.02.010 16644054

[B70] Gómez-Aguado I. Rodríguez-Castejón J. Beraza-Millor M. Vicente-Pascual M. Rodríguez-Gascón A. Garelli S. (2021). Mrna-based nanomedicinal products to address corneal inflammation by interleukin-10 supplementation. Pharmaceutics 13 (9), 1472. 10.3390/pharmaceutics13091472 34575548 PMC8466377

[B71] Gu S. Xing C. Han J. Tso M. O. M. Hong J. (2009). Differentiation of rabbit bone marrow mesenchymal stem cells into corneal epithelial cells *in vivo* and *ex vivo* . Mol. Vis. 15, 99–107. 19156227 PMC2627808

[B72] Guo L. Choi S. Bikkannavar P. Cordeiro M. F. (2022). Microglia: Key players in retinal ageing and neurodegeneration. Front. Cell Neurosci. 16, 804782. 10.3389/fncel.2022.804782 35370560 PMC8968040

[B73] Gupta P. K. Venkateswaran N. (2021). The role of KPI-121 0.25% in the treatment of dry eye disease: Penetrating the mucus barrier to treat periodic flares. Ther. Adv. Ophthalmol. 13, 251584142110127. 10.1177/25158414211012797 PMC8114292 34017938

[B74] Gupta V. Sahu P. K. (2001). Topical cyclosporin A in the management of vernal keratoconjunctivitis. Eye 15 (1), 39–41. 10.1038/eye.2001.10 11318292

[B75] Guter M. Breunig M. (2017). Hyaluronan as a promising excipient for ocular drug delivery. Eur. J. Pharm. Biopharm. 113, 34–49. 10.1016/j.ejpb.2016.11.035 27914235

[B76] Guzman-Aranguez A. Loma P. Pintor J. (2013). Small-interfering RNAs (siRNAs) as a promising tool for ocular therapy. Br. J. Pharmacol. 170 (4), 730–747. 10.1111/bph.12330 23937539 PMC3799589

[B77] Hamrah P. Liu Y. Zhang Q. Dana M. R. (2003). The corneal stroma is endowed with a significant number of resident dendritic cells. Invest. Ophthalmol. Vis. Sci. 44 (2), 581–589. 10.1167/iovs.02-0838 12556386

[B78] Han Y. Xu X. Tang J. Shen C. Lin Q. Chen H. (2017). Bottom-up fabrication of zwitterionic polymer brushes on intraocular lens for improved biocompatibility. Int. J. Nanomedicine 12, 127–135. 10.2147/IJN.S107491 28053528 PMC5191625

[B79] Haneklaus M. Gerlic M. Kurowska-Stolarska M. Rainey A.-A. Pich D. McInnes I. B. (2012). Cutting edge: miR-223 and EBV miR-BART15 regulate the NLRP3 inflammasome and IL-1β production. J. Immunol. 189 (8), 3795–3799. 10.4049/jimmunol.1200312 22984081

[B80] Hao H. He B. Yu B. Yang J. Xing X. Liu W. (2022). Suprachoroidal injection of polyzwitterion hydrogel for treating glaucoma. Biomater. Adv. 142, 213162. SSRN Electronic Journal. 10.1016/j.bioadv.2022.213162 36279749

[B81] He B. Yang J. Liu Y. Xie X. Hao H. Xing X. (2021). An *in situ*-forming polyzwitterion hydrogel: Towards vitreous substitute application. Bioact. Mater 6 (10), 3085–3096. 10.1016/j.bioactmat.2021.02.029 33778190 PMC7960944

[B82] He X. T. Li X. Xia Y. Yin Y. Wu R. X. Sun H. H. (2019). Building capacity for macrophage modulation and stem cell recruitment in high-stiffness hydrogels for complex periodontal regeneration: Experimental studies *in vitro* and in rats. Acta Biomater. 88, 162–180. 10.1016/j.actbio.2019.02.004 30735811

[B83] Herrero-Vanrell R. Bravo-Osuna I. Andrés-Guerrero V. Vicario-de-la-Torre M. Molina-Martínez I. T. (2014). The potential of using biodegradable microspheres in retinal diseases and other intraocular pathologies. Prog. Retin Eye Res. 42, 27–43. 10.1016/j.preteyeres.2014.04.002 24819336

[B84] Hirst S. M. Karakoti A. S. Tyler R. D. Sriranganathan N. Seal S. Reilly C. M. (2009). Anti‐inflammatory properties of cerium oxide nanoparticles. Wiley Online Libr. 5 (24), 2848–2856. 10.1002/smll.200901048 19802857

[B85] Hu C. C. Chiu Y. C. Chaw J. R. Chen C. F. Liu H. W. (2019). Thermo-responsive hydrogel as an anti-VEGF drug delivery system to inhibit retinal angiogenesis in Rex rabbits. Technol. Health Care 27, 153–163. 10.3233/THC-199015 31045535 PMC6597966

[B86] Hu J. Wan Y. (2011). Tolerogenic dendritic cells and their potential applications. Immunology 132 (3), 307. 10.1111/j.1365-2567.2010.03396.x 21208205 PMC3044897

[B87] Huang X. Chau Y. (2021). Enhanced delivery of siRNA to retinal ganglion cells by intravitreal lipid nanoparticles of positive charge. Mol. Pharm. 18 (1), 377–385. 10.1021/acs.molpharmaceut.0c00992 33295773

[B88] Huang X. Chau Y. (2019a). Intravitreal nanoparticles for retinal delivery. Drug Discov. Today 24 (8), 1510–1523. 10.1016/j.drudis.2019.05.005 31102730

[B89] Huang X. Chau Y. (2019b). Investigating impacts of surface charge on intraocular distribution of intravitreal lipid nanoparticles. Exp. Eye Res. 186, 107711. 10.1016/j.exer.2019.107711 31238078

[B90] Hunter R. Strickland F. Kézdy F. (1981). The adjuvant activity of nonionic block polymer surfactants. I. The role of hydrophile-lipophile balance. J. Immunol. 127 (3), 1244–1250. 10.4049/jimmunol.127.3.1244 7264301

[B91] Iezzi R. Guru B. R. Glybina I. v. Mishra M. K. Kennedy A. Kannan R. M. (2012). Dendrimer-based targeted intravitreal therapy for sustained attenuation of neuroinflammation in retinal degeneration. Biomaterials 33 (3), 979–988. 10.1016/j.biomaterials.2011.10.010 22048009

[B92] Im G. (2020). Biomaterials in orthopaedics: The past and future with immune modulation. Biomater. Res. 24 (1), 1–4. 32042442 10.1186/s40824-020-0185-7 PMC7001269

[B93] Ito D. Imai Y. Ohsawa K. Nakajima K. Fukuuchi Y. Kohsaka S. (1998). Microglia-specific localisation of a novel calcium binding protein, Iba1. Mol. Brain Res. 57 (1), 1–9. 10.1016/s0169-328x(98)00040-0 9630473

[B94] Jain E. Hill L. Canning E. Sell S. A. Zustiak S. P. (2017). Control of gelation, degradation and physical properties of polyethylene glycol hydrogels through the chemical and physical identity of the crosslinker. J. Mater Chem. B 5 (14), 2679–2691. 10.1039/c6tb03050e 32264047

[B95] Ji Y. W. Byun Y. J. Choi W. Jeong E. Kim J. S. Noh H. (2013). Neutralization of ocular surface TNF-α reduces ocular surface and lacrimal gland inflammation induced by *in vivo* dry eye. Invest. Ophthalmol. Vis. Sci. 54 (12), 7557–7566. 10.1167/iovs.12-11515 24052636

[B96] Jiang J. O-Bo X. I. A. Hui-Zhuo X. U. Xiong Y. U. Song W. T. Xiong S. I. Q. I. (2009). Inhibition of retinal neovascularization by gene transfer of small interfering RNA targeting HIF-1alpha and VEGF. J. Cell Physiol. 218 (1), 66–74. 10.1002/jcp.21566 18767037

[B97] Kakizawa Y. Lee J. S. Bell B. Fahmy T. M. (2017). Precise manipulation of biophysical particle parameters enables control of proinflammatory cytokine production in presence of TLR 3 and 4 ligands. Acta Biomater. 57, 136–145. 10.1016/j.actbio.2017.01.025 28069499

[B98] Kaplan D. H. Jenison M. C. Saeland S. Shlomchik W. D. Shlomchik M. J. (2005). Epidermal Langerhans cell-deficient mice develop enhanced contact hypersensitivity. Immunity 23 (6), 611–620. 10.1016/j.immuni.2005.10.008 16356859

[B99] Kauper K. Ling V. Elliot S. McGovern C. Sherman S. Dean B. (2012b). Long-term, sustained intraocular delivery of a VEGF antagonist using encapsulated cell technology implant for the treatment of choroidal neovascular diseases. Invest. Ophthalmol. Vis. Sci. 53 (14), 455.

[B100] Kauper K. McGovern C. Sherman S. Heatherton P. Rapoza R. Stabila P. (2012a). Two-year intraocular delivery of ciliary neurotrophic factor by encapsulated cell technology implants in patients with chronic retinal degenerative diseases. Invest. Ophthalmol. Vis. Sci. 53 (12), 7484–7491. 10.1167/iovs.12-9970 23049090

[B101] Kim M. K. Lee J. L. Wee W. R. Lee J. H. (2002). Seoul-type keratoprosthesis: Preliminary results of the first 7 human cases. Archives Ophthalmol. 120 (6), 761–766. 10.1001/archopht.120.6.761 12049581

[B102] Kim T. Sall K. Holland E. J. Brazzell R. K. Coultas S. Gupta P. K. (2018). Safety and efficacy of twice daily administration of KPI-121 1% for ocular inflammation and pain following cataract surgery. Clin. Ophthalmol. 13, 69–86. 10.2147/OPTH.S185800 30643381 PMC6311334

[B103] Kolaczkowska E. Kubes P. (2013). Neutrophil recruitment and function in health and inflammation. Nat. Rev. Immunol. 13 (3), 159–175. 10.1038/nri3399 23435331

[B104] Kong H. J. Alsberg E. Kaigler D. Lee K. Y. Mooney D. J. (2004). Controlling degradation of hydrogels via the size of cross-linked junctions. Adv. Mater 16 (21), 1917–1921. 10.1002/adma.200400014 25067887 PMC4108267

[B105] Kostenko A. Swioklo S. Connon C. J. (2022). Alginate in corneal tissue engineering. Biomed. Mater. 17 (2), 022004. 10.1088/1748-605X/ac4d7b 35051918

[B106] Križaj D. Ryskamp D. A. Tian N. Tezel G. Mitchell C. H. Slepak V. Z. (2014). From mechanosensitivity to inflammatory responses: New players in the pathology of glaucoma. Curr. Eye Res. 39 (2), 105–119. 10.3109/02713683.2013.836541 24144321 PMC3946931

[B107] Kubota I. Sakamoto T. Kawano Y.-I. Tsutsumi T. Hisatomi T. Komiyama S. (2022). Immunoregulatory role of ocular macrophages: The macrophages produce RANTES to suppress experimental autoimmune uveitis. J. Immunol. Ref. 171, 2652–2659. 10.4049/jimmunol.171.5.2652 12928419

[B108] Kutty R. K. Nagineni C. N. Samuel W. Vijayasarathy C. Jaworski C. Duncan T. (2013). Differential regulation of microRNA-146a and microRNA-146b-5p in human retinal pigment epithelial cells by interleukin-1β, tumor necrosis factor-α, and interferon-γ. Mol. Vis. 19, 737–750. 23592910 PMC3626297

[B109] Kwon S. Kim S. H. Khang D. Lee J. Y. (2020). Potential therapeutic usage of nanomedicine for glaucoma treatment. Int. J. Nanomedicine. 15, 5745–5765. 32821099 10.2147/IJN.S254792 PMC7418176

[B110] Kwon C. Kim Y. Jeon H. (2017). Collective migration of lens epithelial cell induced by differential microscale groove patterns. J. Funct. Biomater. 8 (3), 34. 10.3390/jfb8030034 28792434 PMC5618285

[B111] Lajavardi L. Bochot A. Camelo S. Goldenberg B. Naud M. C. Behar-Cohen F. (2007). Downregulation of endotoxin-induced uveitis by intravitreal injection of vasoactive intestinal peptide encapsulated in liposomes. Invest. Ophthalmol. Vis. Sci. 48 (7), 3230–3238. 10.1167/iovs.06-1305 17591893

[B112] Lallemand F. Felt-Baeyens O. Besseghir K. Behar-Cohen F. Gurny R. (2003). Cyclosporine A delivery to the eye: A pharmaceutical challenge. Eur. J. Pharm. Biopharm. 56 (3), 307–318. 10.1016/s0939-6411(03)00138-3 14602172

[B113] Langmann T. (2007). Microglia activation in retinal degeneration. J. Leukoc. Biol. 81 (6), 1345–1351. 10.1189/jlb.0207114 17405851

[B114] Lau C. M. L. Jahanmir G. Yu Y. Chau Y. (2021). Controllable multi-phase protein release from *in-situ* hydrolyzable hydrogel. J. Control. Release 335, 75–85. 10.1016/j.jconrel.2021.05.006 33971140

[B115] Lechner J. Chen M. Hogg R. E. Toth L. Silvestri G. Chakravarthy U. (2017). Peripheral blood mononuclear cells from neovascular age-related macular degeneration patients produce higher levels of chemokines CCL2 (MCP-1) and CXCL8 (IL-8). J. Neuroinflammation 14 (1), 42. 10.1186/s12974-017-0820-y 28231837 PMC5324243

[B116] Lee H. J. Ko J. H. Jeong H. J. Ko A. Y. Kim M. K. Wee W. R. (2015). Mesenchymal stem/stromal cells protect against autoimmunity via CCL2-dependent recruitment of myeloid-derived suppressor cells. J. Immunol. 194 (8), 3634–3645. 10.4049/jimmunol.1402139 25769927

[B117] Li H. Yang Y. G. Sun T. (2022). Nanoparticle-based drug delivery systems for induction of tolerance and treatment of autoimmune diseases. Front. Bioeng. Biotechnol. 10, 889291. 10.3389/fbioe.2022.889291 35464732 PMC9019755

[B118] Li J. Jiang X. Li H. Gelinsky M. Gu Z. Li J. (2021). Tailoring materials for modulation of macrophage fate. Adv. Mater. 33 (12), e2004172. 10.1002/adma.202004172 33565154 PMC9245340

[B119] Li N. Zhuang C. Wang M. Sun X. Nie S. Pan W. (2009). Liposome coated with low molecular weight chitosan and its potential use in ocular drug delivery. Int. J. Pharm. 379 (1), 131–138. 10.1016/j.ijpharm.2009.06.020 19559775

[B120] Li Y. Ren X. Zhang Z. Duan Y. Li H. Chen S. (2022). Effect of small extracellular vesicles derived from IL-10-overexpressing mesenchymal stem cells on experimental autoimmune uveitis. Stem Cell Res. Ther. 13 (1), 100–115. 10.1186/s13287-022-02780-9 35255957 PMC8900327

[B121] Li Y. Y. Cui J. G. Dua P. Pogue A. I. Bhattacharjee S. Lukiw W. J. (2011). Differential expression of miRNA-146a-regulated inflammatory genes in human primary neural, astroglial and microglial cells. Neurosci. Lett. 499 (2), 109–113. 10.1016/j.neulet.2011.05.044 21640790 PMC3713470

[B122] Li Z. Bratlie K. M. (2021). Effect of RGD functionalization and stiffness of gellan gum hydrogels on macrophage polarization and function. Mater. Sci. Eng. C 128, 112303. 10.1016/j.msec.2021.112303 34474854

[B123] Liew P. X. Kubes P. (2019). The neutrophil’s role during health and disease. Physiol. Rev. 99 (2), 1223–1248. 10.1152/physrev.00012.2018 30758246

[B124] Lin H. Yue Y. Maidana D. E. Bouzika P. Atik A. Matsumoto H. (2016). Drug delivery nanoparticles: Toxicity comparison in retinal pigment epithelium and retinal vascular endothelial cells. Semin. Ophthalmol. 31, 1–9. 10.3109/08820538.2015.1114865 26959123 PMC5405708

[B125] Lin W. Liu T. Wang B. Bi H. (2019). The role of ocular dendritic cells in uveitis. Immunol. Lett. 209, 4–10. 10.1016/j.imlet.2019.03.016 30926373

[B126] Liu J. Li Z. (2021). Resident innate immune cells in the cornea. Front. Immunol. 12, 620284. 10.3389/fimmu.2021.620284 33717118 PMC7953153

[B127] Lopez P. F. Grossniklaus H. E. Lambert H. M. Aaberg T. M. Capone A. Sternberg P. (1991). Pathologic features of surgically excised subretinal neovascular membranes in age-related macular degeneration. Am. J. Ophthalmol. 112 (6), 647–656. 10.1016/s0002-9394(14)77270-8 1957899

[B128] Lu J. M. Song X. J. Wang H. F. Li X. L. Zhang X. R. (2012). Murine corneal stroma cells inhibit LPS-induced dendritic cell maturation partially through TGF-β2 secretion *in vitro* . Mol. Vis. 18, 2255–2264. 22933838 PMC3429355

[B129] Luis D. Kieran F. Marc F.-Y. Manus B. Abhay P. Dimitrios Z. (2016). Collagen cross-linking increases scaffold stability while modulates pro-inflammatory macrophage response. Front. Bioeng. Biotechnol. 4. 10.3389/conf.fbioe.2016.01.02202

[B130] Lukiw W. J. Surjyadipta B. Dua P. Alexandrov P. N. (2012). Common micro RNAs (miRNAs) target complement factor H (CFH) regulation in Alzheimer’s disease (AD) and in age-related macular degeneration (AMD). Int. J. Biochem. Mol. Biol. 3 (1), 105–116. 22509485 PMC3325769

[B131] Luo L. J. Nguyen D. D. Lai J. Y. (2020). Dually functional hollow ceria nanoparticle platform for intraocular drug delivery: A push beyond the limits of static and dynamic ocular barriers toward glaucoma therapy. Biomaterials 243, 119961. 10.1016/j.biomaterials.2020.119961 32171102

[B132] Luo L. Yang J. Oh Y. Hartsock M. J. Xia S. Kim Y. C. (2019). Controlled release of corticosteroid with biodegradable nanoparticles for treating experimental autoimmune uveitis. J. Control. Release 296, 68–80. 10.1016/j.jconrel.2019.01.018 30660629 PMC6476551

[B133] Lutty G. A. Cao J. McLeod D. S. (1997). Relationship of polymorphonuclear leukocytes to capillary dropout in the human diabetic choroid. Am. J. Pathology 151 (3), 707–714. PMC1857840 9284819

[B134] Mahajan A. Hasíková L. Hampel U. Grüneboom A. Shan X. Herrmann I. (2021). Aggregated neutrophil extracellular traps occlude Meibomian glands during ocular surface inflammation. Ocul. Surf. 20, 1–12. 10.1016/j.jtos.2020.12.005 33401018

[B135] Mahaling B. Low S. W. Y. Beck M. Kumar D. Ahmed S. Connor T. B. (2022). Damage-associated molecular patterns (DAMPs) in retinal disorders. Int. J. Mol. Sci. 23 (5), 2591. 10.3390/ijms23052591 35269741 PMC8910759

[B136] Mandal A. Pal D. Agrahari V. My Trinh H. Joseph M. Mitra A. K. (2018). Ocular delivery of proteins and peptides: Challenges and novel formulation approaches. Adv. Drug Deliv. Rev. 126, 67–95. 10.1016/j.addr.2018.01.008 29339145 PMC5995646

[B137] Marõ´a M. Alonso J. Sá Nchez A. Alonso M. J. Mar|´a M. Sá A. (2003). The potential of chitosan in ocular drug delivery. Wiley Online Libr. 55 (11), 1451–1463. 10.1211/0022357022476 14713355

[B138] Maruoka S. Inaba M. Ogata N. (2018). Activation of dendritic cells in dry eye mouse model. Invest. Ophthalmol. Vis. Sci. 59 (8), 3269–3277. 10.1167/iovs.17-22550 29971446

[B139] Mathurm M. Gilhotra R. M. (2011). Glycerogelatin-based ocular inserts of aceclofenac: Physicochemical, drug release studies and efficacy against prostaglandin E₂-induced ocular inflammation. Drug Deliv. 18 (1), 54–64. 10.3109/10717544.2010.509366 20718601

[B140] Matica M. A. Aachmann F. L. Tøndervik A. Sletta H. Ostafe V. (2019). Chitosan as a wound dressing starting material: Antimicrobial properties and mode of action. Int. J. Mol. Sci. 20 (23), 5889. 10.3390/ijms20235889 31771245 PMC6928789

[B141] McMenamin P. G. Saban D. R. Dando S. J. (2019). Immune cells in the retina and choroid: Two different tissue environments that require different defenses and surveillance. Prog. Retin Eye Res. 70, 85–98. 10.1016/j.preteyeres.2018.12.002 30552975 PMC7321801

[B142] McWhorter F. Y. Wang T. Nguyen P. Chung T. Liu W. F. (2013). Modulation of macrophage phenotype by cell shape. Proc. Natl. Acad. Sci. U. S. A. 110 (43), 17253–17258. 10.1073/pnas.1308887110 24101477 PMC3808615

[B143] Medawar P. B. (1948). Immunity to homologous grafted skin; the fate of skin homografts transplanted to the brain, to subcutaneous tissue, and to the anterior chamber of the eye. Br. J. Exp. Pathol. 29 (1), 58–69. 18865105 PMC2073079

[B144] Merad M. Ginhoux F. Collin M. (2008). Origin, homeostasis and function of Langerhans cells and other langerin-expressing dendritic cells. Nat. Rev. Immunol. 8 (12), 935–947. 10.1038/nri2455 19029989

[B145] Mérida S. Palacios E. Navea A. Bosch-Morell F. (2015). Macrophages and uveitis in experimental animal models. Mediat. Inflamm. 2015, 671417. 10.1155/2015/671417 PMC4452861 26078494

[B146] Minghetti L. Malchiodi-Albedi F. Matteucci A. Bernardo A. (2008). PPAR-γ, microglial cells, and ocular inflammation: New venues for potential therapeutic approaches. PPAR Res. 2008, 295784. 10.1155/2008/295784 18382616 PMC2276614

[B147] Mitarotonda R. Giorgi E. Eufrasio-da-Silva T. Dolatshahi-Pirouz A. Mishra Y. K. Khademhosseini A. (2022). Immunotherapeutic nanoparticles: From autoimmune disease control to the development of vaccines. Biomater. Adv. 135, 212726. 10.1016/j.bioadv.2022.212726 35475005 PMC9023085

[B148] Mobaraki M. Soltani M. Harofte S. Z. Zoudani E. L. Daliri R. Aghamirsalim M. (2020). Pharmaceutics biodegradable nanoparticle for cornea drug delivery: Focus review. Pharmaceutics 12, 1232. 10.3390/pharmaceutics12121232 33353013 PMC7765989

[B149] Morante-Palacios O. Fondelli F. Ballestar E. Martínez-Cáceres E. M. (2021). Tolerogenic dendritic cells in autoimmunity and inflammatory diseases. Trends Immunol. 42 (1), 59–75. 10.1016/j.it.2020.11.001 33293219

[B150] Mori K. Gehlbach P. Yamamoto S. Duh E. Zack D. J. Li Q. (2002). AAV-mediated gene transfer of pigment epithelium-derived factor inhibits choroidal neovascularization. Invest. Ophthalmol. Vis. Sci. 43 (6), 1994–2000. 12037010

[B151] Morillas A. G. Besson V. C. Lerouet D. Aranda C. (2021). Molecular sciences microglia and neuroinflammation: What place for P2RY12? Int. J. Mol. Sci. 22 (4), 1636. 10.3390/ijms22041636 33561958 PMC7915979

[B152] Moshaverinia A. Chen C. Xu X. Ansari S. Zadeh H. H. Schricker S. R. (2015). Regulation of the stem cell–host immune system interplay using hydrogel coencapsulation system with an anti‐inflammatory drug. Adv. Funct. Mat. 25 (15), 2296–2307. Wiley Online Library. 10.1002/adfm.201500055 PMC4478611 26120294

[B153] Mosser D. M. Edwards J. P. (2008). Exploring the full spectrum of macrophage activation. Nat. Rev. Immunol. 8 (12), 958–969. 10.1038/nri2448 19029990 PMC2724991

[B154] Mun Y. Hwang J. S. Shin Y. J. (2021). Role of neutrophils on the ocular surface. Int. J. Mol. Sci. 22 (19), 10386. 10.3390/ijms221910386 34638724 PMC8508808

[B155] Murakami Y. Ishikawa K. Nakao S. Sonoda K. H. (2020). Innate immune response in retinal homeostasis and inflammatory disorders. Prog. Retin Eye Res. 74, 100778. 10.1016/j.preteyeres.2019.100778 31505218

[B156] Musumeci A. Lutz K. Winheim E. Krug A. B. (2019). What makes a PDC: Recent advances in understanding plasmacytoid DC development and heterogeneity. Front. Immunol. 10, 1222. 10.3389/fimmu.2019.01222 31191558 PMC6548821

[B157] Nickerson C. S. Karageozian H. L. Park J. Kornfield J. A. (2004). The mechanical properties of the vitreous humor. Invest. Ophthalmol. Vis. Sci. 45 (13), 37.

[B158] Niederkorn J. Y. (2019). The eye sees eye to eye with the immune system: The 2019 proctor lecture. Invest. Ophthalmol. Vis. Sci. 60 (13), 4489–4495. 10.1167/iovs.19-28632 31661549 PMC6819053

[B159] Niikura K. Matsunaga T. Suzuki T. Kobayashi S. Yamaguchi H. Orba Y. (2013). Gold nanoparticles as a vaccine platform: Influence of size and shape on immunological responses *in vitro* and *in vivo* . ACS Nano 7 (5), 3926–3938. 10.1021/nn3057005 23631767

[B160] Nussenblatt R. B. Palestine A. G. (1986). Cyclosporine: Immunology, pharmacology and therapeutic uses. Surv. Ophthalmol. 31 (3), 159–169. 10.1016/0039-6257(86)90035-4 3544293

[B161] Oh J. Y. Lee R. H. (2021). Mesenchymal stromal cells for the treatment of ocular autoimmune diseases. Prog. Retin Eye Res. 85, 100967. 10.1016/j.preteyeres.2021.100967 33775824 PMC8922475

[B162] Ohsawa K. Imai Y. Kanazawa H. Sasaki Y. Kohsaka S. (2000). Involvement of Iba1 in membrane ruffling and phagocytosis of macrophages/microglia. J. Cell Sci. 113 (17), 3073–3084. 10.1242/jcs.113.17.3073 10934045

[B163] Orozco Morales M. L. Marsit N. M. McIntosh O. D. Hopkinson A. Sidney L. E. (2019). Anti-inflammatory potential of human corneal stroma-derived stem cells determined by a novel *in vitro* corneal epithelial injury model. World J. Stem Cells 11 (2), 84–99. 10.4252/wjsc.v11.i2.84 30842807 PMC6397805

[B164] Østerholt H. Lundeland B. S. Sonerud T. Saugstad O. D. Nakstad B. (2011). Effects of hyaluronic acid on expression of TLR2 and TLR4 on cord blood monocytes. Pediatr. Res. 70 (5), 476. 10.1038/pr.2011.701

[B165] Papenburg B. J. Rodrigues E. D. Wessling M. Stamatialis D. (2010). Insights into the role of material surface topography and wettability on cell-material interactions. Soft Matter 6 (18), 4377–4388. 10.1039/b927207k

[B166] Park K. Chen Y. Hu Y. Mayo A. S. Kompella U. B. Longeras R. (2009). Nanoparticle-mediated expression of an angiogenic inhibitor ameliorates ischemia-induced retinal neovascularization and diabetes-induced retinal vascular leakage. Diabetes 58 (8), 1902–1913. 10.2337/db08-1327 19491211 PMC2712783

[B167] Parolini O. Soncini M. Evangelista M. Schmidt D. (2009). Amniotic membrane and amniotic fluid-derived cells: Potential tools for regenerative medicine? Regen. Med. 4 (2), 275–291. 10.2217/17460751.4.2.275 19317646

[B168] Penfold P. L. Madigan M. C. Gillies M. C. Provis J. M. (2001). Immunological and aetiological aspects of macular degeneration. Prog. Retin Eye Res. 20 (3), 385–414. 10.1016/s1350-9462(00)00025-2 11286898

[B169] Pereira D. V. Petronilho F. Pereira H. R. S. B. Vuolo F. Mina F. Possato J. C. (2012). Effects of gold nanoparticles on endotoxin-induced uveitis in rats. Invest. Ophthalmol. Vis. Sci. 53 (13), 8036–8041. 10.1167/iovs.12-10743 23150627

[B170] Perez V. L. Caspi R. R. (2015). Immune mechanisms in inflammatory and degenerative eye disease. Trends Immunol. 36 (6), 354–363. 10.1016/j.it.2015.04.003 25981967 PMC4563859

[B171] Peynshaert K. Devoldere J. de Smedt S. C. Remaut K. (2018). *In vitro* and *ex vivo* models to study drug delivery barriers in the posterior segment of the eye. Adv. Drug Deliv. Rev. 126, 44–57. 10.1016/j.addr.2017.09.007 28939376

[B172] Peynshaert K. Devoldere J. Minnaert A. K. de Smedt S. C. Remaut K. (2019). Morphology and composition of the inner limiting membrane: Species-specific variations and relevance toward drug delivery research. Drug Deliv. Res. 44 (5), 465–475. 10.1080/02713683.2019.1565890 30638413

[B173] Pillay J. den Braber I. Vrisekoop N. Kwast L. M. de Boer R. J. Borghans J. A. M. (2010). *In vivo* labeling with 2H2O reveals a human neutrophil lifespan of 5.4 days. Blood 116 (4), 625–627. 10.1182/blood-2010-01-259028 20410504

[B174] Pintor J. (2012). Silencing beta2-adrenergic receptors reduces intraocular pressure: A new approach for glaucoma therapy: A new approach to glaucoma therapy. Ann. R. Natl. Acad. Pharm. 78 (2), 230–240.

[B175] Pintwala R. Postnikoff C. Molladavoodi S. Gorbet M. (2014). Coculture with intraocular lens material-activated macrophages induces an inflammatory phenotype in lens epithelial cells. J. Biomater. Appl. 29 (8), 1119–1132. 10.1177/0885328214552711 25281645

[B176] Pogue A. I. Lukiw W. J. (2018). Up-regulated pro-inflammatory MicroRNAs (miRNAs) in alzheimer’s disease (AD) and age-related macular degeneration (AMD). Cell Mol. Neurobiol. 38 (5), 1021–1031. 10.1007/s10571-017-0572-3 29302837 PMC11481951

[B177] Puri S. Kenyon B. M. Hamrah P. (2022). Immunomodulatory role of neuropeptides in the cornea. Biomedicines 10 (8), 1985. 10.3390/biomedicines10081985 36009532 PMC9406019

[B178] Raghunath A. Perumal E. (2015). Micro-RNAs and their roles in eye disorders. Ophthalmic Res. 53 (4), 169–186. 10.1159/000371853 25832915

[B179] Ramírez A. I. de Hoz R. Fernández-Albarral J. A. Salobrar-Garcia E. Rojas B. Valiente-Soriano F. J. (2020). Time course of bilateral microglial activation in a mouse model of laser-induced glaucoma. Sci. Rep. 10 (1), 4890. 10.1038/s41598-020-61848-9 32184450 PMC7078298

[B180] Rathod S. Deshpande S. (2008). Albumin microspheres as an ocular delivery system for pilocarpine nitrate. Indian J. Pharm. Sci. 70 (2), 193–197. 10.4103/0250-474X.41454 20046711 PMC2792486

[B181] Rayahin J. E. Buhrman J. S. Zhang Y. Koh T. J. Gemeinhart R. A. (2015). High and low molecular weight hyaluronic acid differentially influence macrophage activation. ACS Biomater. Sci. Eng. 1 (7), 481–493. 10.1021/acsbiomaterials.5b00181 26280020 PMC4533115

[B182] Reeves A. R. D. Spiller K. L. Freytes D. O. Vunjak-Novakovic G. Kaplan D. L. (2015). Controlled release of cytokines using silk-biomaterials for macrophage polarization. Biomaterials 73, 272–283. 10.1016/j.biomaterials.2015.09.027 26421484 PMC4605898

[B183] Reich S. Fosnot J. Kuroki A. Tang W. Vis X. Y.-M. (2003). Small interfering RNA (siRNA) targeting VEGF effectively inhibits ocular neovascularization in a mouse model. Mol. Vis. 9, 210–216. 12789138

[B184] Reichenbach A. Bringmann A. (2020). Glia of the human retina. Glia 68 (4), 768–796. 10.1002/glia.23727 31793693

[B185] Reid B. Gibson M. Singh A. Taube J. Furlong C. Murcia M. (2015). PEG hydrogel degradation and the role of the surrounding tissue environment. J. Tissue Eng. Regen. Med. 9 (3), 315–318. 10.1002/term.1688 23495204 PMC4819972

[B186] Ren N. Sun R. Xia K. Zhang Q. Li W. Wang F. (2019). DNA-based hybrid hydrogels sustain water-insoluble ophthalmic therapeutic delivery against allergic conjunctivitis. ACS Appl. Mater Interfaces 11 (30), 26704–26710. 10.1021/acsami.9b08652 31264833

[B187] Riabov V. Salazar F. Htwe S. S. Gudima A. Schmuttermaier C. Barthes J. (2017). Generation of anti-inflammatory macrophages for implants and regenerative medicine using self-standing release systems with a phenotype-fixing cytokine cocktail formulation. Acta Biomater. 53, 389–398. 10.1016/j.actbio.2017.01.071 28159717

[B188] Ron E. Turek T. Mathiowitz E. Chasin M. Hageman M. Langer R. (1993). Controlled release of polypeptides from polyanhydrides. Proc. Natl. Acad. Sci. U. S. A. 90 (9), 4176–4180. 10.1073/pnas.90.9.4176 8483931 PMC46469

[B189] Rota R. Riccioni T. Zaccarini M. Lamartina S. del Gallo A. Fusco A. (2004). Marked inhibition of retinal neovascularization in rats following soluble-flt-1 gene transfer. J. Gene Med. 6 (9), 992–1002. 10.1002/jgm.586 15352072

[B190] Rowley A. T. Nagalla R. R. Wang S. W. Liu W. F. (2019). Extracellular matrix-based strategies for immunomodulatory biomaterials engineering. Adv. Healthc. Mater 8 (8), e1801578. 10.1002/adhm.201801578 30714328 PMC7568845

[B191] Rumelt S. Bersudsky V. Blum-Hareuveni T. Rehany U. (2002). Systemic cyclosporin A in high failure risk, repeated corneal transplantation. Br. J. Ophthalmol. 86 (9), 988–992. 10.1136/bjo.86.9.988 12185123 PMC1771260

[B192] Russell S. Bennett J. Wellman J. A. Chung D. C. Yu Z. F. Tillman A. (2017). Efficacy and safety of voretigene neparvovec (AAV2-hRPE65v2) in patients with RPE65-mediated inherited retinal dystrophy: A randomised, controlled, open-label, phase 3 trial. Lancet 390 (10097), 849–860. 10.1016/S0140-6736(17)31868-8 28712537 PMC5726391

[B193] Ryoo N. K. Lee J. Lee H. Hong H. K. Kim H. Lee J. B. (2017). Therapeutic effects of a novel siRNA-based anti-VEGF (siVEGF) nanoball for the treatment of choroidal neovascularization. Nanoscale 9 (40), 15461–15469. 10.1039/c7nr03142d 28976519

[B194] Sakai T. Ishihara T. Higaki M. Akiyama G. Tsuneoka H. (2011). Therapeutic effect of stealth-type polymeric nanoparticles with encapsulated betamethasone phosphate on experimental autoimmune uveoretinitis. Invest. Ophthalmol. Vis. Sci. 52 (3), 1516–1521. 10.1167/iovs.10-5676 21178146

[B195] Sakai T. Kohno H. Ishihara T. Higaki M. Saito S. Matsushima M. (2006). Treatment of experimental autoimmune uveoretinitis with poly(lactic acid) nanoparticles encapsulating betamethasone phosphate. Exp. Eye Res. 82 (4), 657–663. 10.1016/j.exer.2005.09.003 16360654

[B196] Schanen B. C. Das S. Reilly C. M. Warren W. L. Self W. T. Seal S. (2013). Immunomodulation and T helper TH₁/TH₂ response polarization by CeO₂ and TiO₂ nanoparticles. PLoS One 8 (5), e62816. 10.1371/journal.pone.0062816 23667525 PMC3648566

[B197] Schirmer L. Atallah P. Werner C. Freudenberg U. (2016). StarPEG-heparin hydrogels to protect and sustainably deliver IL-4. Adv. Healthc. Mater 5 (24), 3157–3164. 10.1002/adhm.201600797 27860466

[B198] Schopf L. R. Popov A. M. Enlow E. M. Bourassa J. L. Ong W. Z. Nowak P. (2015). Topical ocular drug delivery to the back of the eye by mucus-penetrating particles. Transl. Vis. Sci. Technol. 4 (3), 11. 10.1167/tvst.4.3.11 PMC4473722 26101724

[B199] Schroder S. Palinski W. Schmid-Schonbein G. W. (1991). Activated monocytes and granulocytes, capillary nonperfusion, and neovascularization in diabetic retinopathy. Am. J. Pathology 139 (1), 81–100. PMC1886150 1713023

[B200] Seong S. Y. Matzinger P. (2004). Hydrophobicity: An ancient damage-associated molecular pattern that initiates innate immune responses. Nat. Rev. Immunol. 4 (6), 469–478. 10.1038/nri1372 15173835

[B201] Serda M. Becker F. G. Cleary M. Team R. M. Holtermann H. The D. (1990). Update on topical cyclosporin A: Background, immunology, and pharmacology. Cornea 9 (3), 184–195. 10.1097/00003226-199007000-00002 2197063

[B202] Shafie M. Fayek H. (2013). Formulation and evaluation of betamethasone sodium phosphate loaded nanoparticles for ophthalmic delivery. J. Clin. Exp. Ophthalmol. 4, 273. 10.4172/2155-9570.1000273

[B203] Sharma A. K. Arya A. Sahoo P. K. Majumdar D. K. (2016). Overview of biopolymers as carriers of antiphlogistic agents for treatment of diverse ocular inflammations. Mater. Sci. Eng. C 67, 779–791. 10.1016/j.msec.2016.05.060 27287177

[B204] Shaunak S. Thomas S. Gianasi E. Godwin A. Jones E. Teo I. (2004). Polyvalent dendrimer glucosamine conjugates prevent scar tissue formation. Nat. Biotechnol. 22 (8), 977–984. 10.1038/nbt995 15258595

[B205] Shen J. Samul R. Silva R. L. Akiyama H. Liu H. Saishin Y. (2006). Suppression of ocular neovascularization with siRNA targeting VEGF receptor 1. Gene Ther. 13 (3), 225–234. 10.1038/sj.gt.3302641 16195704

[B206] Shi H. Ding J. Chen C. Yao Q. Zhang W. Fu Y. (2021). Antimicrobial action of biocompatible silver microspheres and their role in the potential treatment of fungal keratitis. ACS Biomater. Sci. Eng. 7 (11), 5090–5098. 10.1021/acsbiomaterials.1c00815 34634199

[B207] Silva D. Pinto L. F. V. Bozukova D. Santos L. F. Serro A. P. Saramago B. (2016). Chitosan/alginate based multilayers to control drug release from ophthalmic lens. Colloids Surf. B Biointerfaces 147, 81–89. 10.1016/j.colsurfb.2016.07.047 27494772

[B208] Simpson F. C. McTiernan C. D. Islam M. M. Buznyk O. Lewis P. N. Meek K. M. (2021). Collagen analogs with phosphorylcholine are inflammation-suppressing scaffolds for corneal regeneration from alkali burns in mini-pigs. Commun. Biol. 4, 608. 10.1038/s42003-021-02108-y 34021240 PMC8140136

[B209] Singh R. Batoki J. C. Ali M. Bonilha V. L. Anand-Apte B. (2020). Inhibition of choroidal neovascularization by systemic delivery of gold nanoparticles. Nanomedicine 28, 102205. 10.1016/j.nano.2020.102205 32305594 PMC7438302

[B210] Soiberman U. Kambhampati S. P. Wu T. Mishra M. K. Oh Y. Sharma R. (2017). Subconjunctival injectable dendrimer-dexamethasone gel for the treatment of corneal inflammation. Biomaterials 125, 38–53. 10.1016/j.biomaterials.2017.02.016 28226245 PMC5870122

[B211] Song H. B. Park S. Y. Ko J. H. Park J. W. Yoon C. H. Kim D. H. (2018). Mesenchymal stromal cells inhibit inflammatory lymphangiogenesis in the cornea by suppressing macrophage in a TSG-6-dependent manner. Mol. Ther. 26 (1), 162–172. 10.1016/j.ymthe.2017.09.026 29301108 PMC5763076

[B212] Song J. Winkeljann B. Lieleg O. (2020). Biopolymer-based coatings: Promising strategies to improve the biocompatibility and functionality of materials used in biomedical engineering. Adv. Mater Interfaces 7 (17), 2000850. 10.1002/admi.202000850

[B213] Song Y. Overmass M. Fan J. Hodge C. Sutton G. Lovicu F. J. (2021). Application of collagen I and IV in bioengineering transparent ocular tissues. Front. Surg. 8, 639500. 10.3389/fsurg.2021.639500 34513910 PMC8427501

[B214] Sonoda K.-H. Sasa Y. Qiao H. Tsutsumi C. Hisatomi T. Komiyama S. (2003). Immunoregulatory role of ocular macrophages: The macrophages produce RANTES to suppress experimental autoimmune uveitis. J. Immunol. 171 (5), 2652–2659. 10.4049/jimmunol.171.5.2652 12928419

[B215] Souto E. B. Dias-Ferreira J. López-Machado A. Ettcheto M. Cano A. Espuny A. C. (2019). Advanced formulation approaches for ocular drug delivery: State-Of-The-Art and recent patents. Pharmaceutics 11 (9), 460. 10.3390/pharmaceutics11090460 31500106 PMC6781321

[B216] Sridharan R. Cavanagh B. Cameron A. R. Kelly D. J. O’Brien F. J. (2019). Material stiffness influences the polarization state, function and migration mode of macrophages. Acta Biomater. 89, 47–59. 10.1016/j.actbio.2019.02.048 30826478

[B217] Sugita S. Kawazoe Y. Imai A. Usui Y. Iwakura Y. Isoda K. (2013). Mature dendritic cell suppression by IL-1 receptor antagonist on retinal pigment epithelium cells. Invest. Ophthalmol. Vis. Sci. 54 (5), 3240–3249. 10.1167/iovs.12-11483 23532521

[B218] Sugita S. Usui Y. Horie S. Futagami Y. Aburatani H. Okazaki T. (2009). T-cell suppression by programmed cell death 1 ligand 1 on retinal pigment epithelium during inflammatory conditions. Invest. Ophthalmol. Vis. Sci. 50 (6), 2862–2870. 10.1167/iovs.08-2846 19182257

[B219] Tamura T. Ishikawa N. Tanaka S. Hayashi Y. Miyamoto T. Saika S. (2017). Histopathological analyses of the differences in foreign body cell reactions against intraocular lenses according to the period of implantation. J. Eye Cataract Surg. 3. 10.21767/2471-8300.100017

[B220] Tan W. Zou J. Yoshida S. Jiang B. Zhou Y. (2020). The role of inflammation in age-related macular degeneration. Int. J. Biol. Sci. 16 (15), 2989–3001. 10.7150/ijbs.49890 33061811 PMC7545698

[B221] Tang Z. Fan X. Chen Y. Gu P. (2022). Ocular nanomedicine. Adv. Sci. 9 (15), 2003699. 10.1002/advs.202003699 PMC9130902 35150092

[B222] Taskin M. B. Tylek T. Blum C. Böhm C. Wiesbeck C. Groll J. (2021). Inducing immunomodulatory effects on human macrophages by multifunctional NCO-sP(EO-*stat*-PO)/Gelatin hydrogel nanofibers. ACS Biomater. Sci. Eng. 7 (7), 3166–3178. 10.1021/acsbiomaterials.1c00232 34114792

[B223] Taylor A. W. Ng T. F. (2018). Negative regulators that mediate ocular immune privilege. J. Leukoc. Biol. 103 (6), 1179–1187. 10.1002/JLB.3MIR0817-337R PMC6240388 29431864

[B224] Taylor A. W. (2016). Ocular immune privilege and transplantation. Front. Immunol. 7, 37. 10.3389/fimmu.2016.00037 26904026 PMC4744940

[B225] Taylor A. W. (2007). Ocular immunosuppressive microenvironment. Chem. Immunol. Allergy 92, 71–85. 10.1159/000099255 17264484

[B226] Timmers A. M. Newmark J. A. Turunen H. T. Farivar T. Liu J. Song C. (2020). Ocular inflammatory response to intravitreal injection of adeno-associated virus vector: Relative contribution of genome and capsid. Hum. Gene Ther. 31 (1–2), 80–89. 10.1089/hum.2019.144 31544533

[B227] Tisi A. Passacantando M. Lozzi L. Riccitelli S. Bisti S. Maccarone R. (2019). Retinal long term neuroprotection by Cerium Oxide nanoparticles after an acute damage induced by high intensity light exposure. Exp. Eye Res. 182, 30–38. 10.1016/j.exer.2019.03.003 30867118

[B228] Tiwari R. Sethiya N. K. Gulbake A. S. Mehra N. K. Murty U. S. N. Gulbake A. (2021). A review on albumin as a biomaterial for ocular drug delivery. Int. J. Biol. Macromol. 191, 591–599. 10.1016/j.ijbiomac.2021.09.112 34562538

[B229] Toscano M. A. Commodaro A. G. Ilarregui J. M. Bianco G. A. Liberman A. Serra H. M. (2006). Galectin-1 suppresses autoimmune retinal disease by promoting concomitant Th2- and T regulatory-mediated anti-inflammatory responses. J. Immunol. 176 (10), 6323–6332. 10.4049/jimmunol.176.10.6323 16670344

[B230] Trattler W. Hosseini K. (2017). Twice-daily vs. Once-daily dosing with 0.075% bromfenac in DuraSite: Outcomes from a 14-day phase 2 study. Ophthalmol. Ther. 6 (2), 277–284. 10.1007/s40123-017-0102-x 28819932 PMC5693819

[B231] Tsai C. H. Wang P. Y. Lin I. C. Huang H. Liu G. S. Tseng C. L. (2018). Ocular drug delivery: Role of degradable polymeric nanocarriers for ophthalmic application. Int. J. Mol. Sci. 19, 2830. 10.3390/ijms19092830 30235809 PMC6164366

[B232] Tseng C. L. Chen K. H. Su W. Y. Lee Y. H. Wu C. C. Lin F. H. (2013). Cationic gelatin nanoparticles for drug delivery to the ocular surface: *In vitro* and *in vivo* evaluation. J. Nanomater 2013, 1–11. 10.1155/2013/238351

[B233] Tsukamoto T. Hironaka K. Fujisawa T. Yamaguchi D. Tahara K. Tozuka Y. (2013). Preparation of bromfenac-loaded liposomes modified with chitosan for ophthalmic drug delivery and evaluation of physicochemical properties and drug release profile. Asian J. Pharm. Sci. 8 (2), 104–109. 10.1016/j.ajps.2013.07.013

[B234] van Dooremaal J. C. (1873). Die Entwicklung der in fremden grund versetzten lebenden geweba. Albr. Graefes Arch Ophthalmol 19, 358–373.

[B235] Vandervoort J. Ludwig A. (2004). Preparation and evaluation of drug-loaded gelatin nanoparticles for topical ophthalmic use. Eur. J. Pharm. Biopharm. 57 (2), 251–261. 10.1016/S0939-6411(03)00187-5 15018982

[B236] Vassey M. J. Figueredo G. P. Scurr D. J. Vasilevich A. S. Vermeulen S. Carlier A. (2020). Immune modulation by design: Using topography to control human monocyte attachment and macrophage differentiation. Adv. Sci. 7 (11), 1903392. 10.1002/advs.201903392 PMC7284204 32537404

[B237] Veiseh O. Vegas A. J. (2019). Domesticating the foreign body response: Recent advances and applications. Adv. Drug Deliv. Rev. 144, 148–161. 10.1016/j.addr.2019.08.010 31491445 PMC6774350

[B238] Verbeke C. S. Mooney D. J. Verbeke C. S. Mooney John A Paulson D. J. Mooney D. J. (2015). Injectable, pore-forming hydrogels for *in vivo* enrichment of immature dendritic cells. Adv. Healthc. Mater 4 (17), 2677–2687. 10.1002/adhm.201500618 26474318 PMC4715727

[B239] Wakefield D. Gray P. Chang J. di Girolamo N. McCluskey P. (2010). The role of PAMPs and DAMPs in the pathogenesis of acute and recurrent anterior uveitis. Br. J. Ophthalmol. 94 (3), 271–274. 10.1136/bjo.2008.146753 19264730

[B240] Wang B. Lin Q. Jin T. Shen C. Tang J. Han Y. (2014). Surface modification of intraocular lenses with hyaluronic acid and lysozyme for the prevention of endophthalmitis and posterior capsule opacification. RSC Adv. 5 (5), 3597–3604. 10.1039/c4ra13499k PMC8982225 35427043

[B241] Wang R. Xia J. Tang J. Liu D. Zhu S. Wen S. (2021). Surface modification of intraocular lens with hydrophilic poly(sulfobetaine methacrylate) brush for posterior capsular opacification prevention. J. Ocul. Pharmacol. Ther. 37 (3), 172–180. 10.1089/jop.2020.0134 33497580

[B242] Wang S. K. Cepko C. L. (2022). Targeting microglia to treat degenerative eye diseases. Front. Immunol. 13, 843558. 10.3389/fimmu.2022.843558 35251042 PMC8891158

[B243] Wasnik V. B. Thool A. R. (2022). Ocular gene therapy: A literature review with focus on current clinical trials. Cureus 14 (9), e29533. 10.7759/cureus.29533 36312652 PMC9590687

[B244] Wen Y. Waltman A. Han H. Collier J. H. (2016). Switching the immunogenicity of peptide assemblies using surface properties. ACS Nano 10 (10), 9274–9286. 10.1021/acsnano.6b03409 27680575 PMC5704984

[B245] Wikström J. Elomaa M. Syväjärvi H. Kuokkanen J. Yliperttula M. Honkakoski P. (2008). Alginate-based microencapsulation of retinal pigment epithelial cell line for cell therapy. Biomaterials 29 (7), 869–876. 10.1016/j.biomaterials.2007.10.056 18045685

[B246] Williams D. F. (1999). The Williams dictionary of biomaterials. Liverpool Univ Press, 343.

[B247] Woźniak A. Malankowska A. Nowaczyk G. Grześkowiak B. F. Tuśnio K. Słomski R. Zaleska-Medynska A. Jurga S. (2017). Size and shape-dependent cytotoxicity profile of gold nanoparticles for biomedical applications. J Mater Sci Mater Med 28 (6), 92. 10.1007/s10856-017-5902-y 28497362

[B248] Wong F. S. Y. Tsang K. K. Lo A. C. Y. (2017). Delivery of therapeutics to posterior eye segment: Cell-encapsulating systems. Neural Regen. Res. 12 (4), 576–577. 10.4103/1673-5374.205093 28553333 PMC5436351

[B249] Wu W. He Z. Zhang Z. Yu X. Song Z. Li X. (2016). Intravitreal injection of rapamycin-loaded polymeric micelles for inhibition of ocular inflammation in rat model. Int. J. Pharm. 513 (1–2), 238–246. 10.1016/j.ijpharm.2016.09.013 27609662

[B250] Xiang J. Sun J. Hong J. Wang W. Wei A. Le Q. (2015). T-style keratoprosthesis based on surface-modified poly (2-hydroxyethyl methacrylate) hydrogel for cornea repairs. Mater. Sci. Eng. C 50, 274–285. 10.1016/j.msec.2015.01.089 25746271

[B251] Xiaonan H. (2020). Engineered nanoparticles for retinal targeted delivery - xiaonan Huang - google books. Hong Kong: Hong Kong University of Science and Technology.

[B252] Yang H. Zheng S. Mao Y. Chen Z. Zheng C. Li H. (2016). Modulating of ocular inflammation with macrophage migration inhibitory factor is associated with notch signalling in experimental autoimmune uveitis. Clin. Exp. Immunol. 183 (2), 280–293. 10.1111/cei.12710 26400205 PMC4711161

[B253] Yavuz B. Bozdağ Pehlivan S. Sümer Bolu B. Nomak Sanyal R. Vural İ. Ünlü N. (2016). Dexamethasone – PAMAM dendrimer conjugates for retinal delivery: Preparation, characterization and *in vivo* evaluation. J. Pharm. Pharmacol. 68 (8), 1010–1020. 10.1111/jphp.12587 27283886

[B254] Yenice İ. Mocan M. Palaska E. Bochotd A. Bilensoy E. Vurala I. (2008). Hyaluronic acid coated poly-ɛ-caprolactone nanospheres deliver high concentrations of cyclosporine A into the cornea. Exp. Eye Res. 87, 162–167. Elsevier. 10.1016/j.exer.2008.04.002 18675411

[B255] Yerramothu P. Vijay A. K. Willcox M. D. P. (2018). Inflammasomes, the eye and anti-inflammasome therapy. Eye 32 (3), 491–505. 10.1038/eye.2017.241 29171506 PMC5848281

[B256] Yıldız M. B. Yıldız E. (2022). Evaluation of serum neutrophil-to-lymphocyte ratio in corneal graft rejection after low-risk penetrating keratoplasty. Int. Ophthalmol. 42 (1), 57–63. 10.1007/s10792-021-01999-4 34387791

[B257] You N. Chu S. Cai B. Gao Y. Hui M. Zhu J. (2020). Bioactive hyaluronic acid fragments inhibit lipopolysaccharide-induced inflammatory responses via the Toll-like receptor 4 signaling pathway. Front. Med. 15 (2), 292–301. 10.1007/s11684-020-0806-5 32946028

[B258] Yu F.-S. X. Hazlett L. D. (2006). Toll-like receptors and the eye. Invest. Ophthalmol. Vis. Sci. 47 (4), 1255–1263. 10.1167/iovs.05-0956 16565355 PMC2666381

[B259] Yu Y. Chow D. W. Y. Lau C. M. L. Zhou G. Back W. Xu J. (2021). A bioinspired synthetic soft hydrogel for the treatment of dry eye. Bioeng. Transl. Med. 6 (3), e10227. 10.1002/btm2.10227 34589602 PMC8459603

[B260] Yu Y. Lau L. C. M. Lo A. C. Chau Y. (2015). Injectable chemically crosslinked hydrogel for the controlled release of bevacizumab in vitreous: A 6-month *in vivo* study. Transl. Vis. Sci. Technol. 4 (2), 5. 10.1167/tvst.4.2.5 PMC4356035 25774331

[B261] Yu Y. Lin X. Wang Q. Chau Y. (2019). Long-term therapeutic effect in nonhuman primate eye from a single injection of anti-VEGF controlled release hydrogel. Bioeng. Transl. Med. 4 (2), e10128. 10.1002/btm2.10128 31249878 PMC6584386

[B262] Zajicova A. Pokorna K. Lencova A. Krulova M. Svobodova E. Kubinova S. (2010). Treatment of ocular surface injuries by limbal and mesenchymal stem cells growing on nanofiber scaffolds. Cell Transpl. 19 (10), 1281–1290. 10.3727/096368910X509040 20573307

[B263] Zhang K. Hopkins J. J. Heier J. S. Birch D. G. Halperin L. S. Albini T. A. (2011). Ciliary neurotrophic factor delivered by encapsulated cell intraocular implants for treatment of geographic atrophy in age-related macular degeneration. Proc. Natl. Acad. Sci. U. S. A. 108 (15), 6241–6245. 10.1073/pnas.1018987108 21444807 PMC3076847

[B264] Zhao Y. Yang K. Li J. Huang Y. Zhu S. (2017). Comparison of hydrophobic and hydrophilic intraocular lens in preventing posterior capsule opacification after cataract surgery: An updated meta-analysis. Medicine 96, e8301. 10.1097/MD.0000000000008301 29095259 PMC5682778

[B265] Zhou Q. Xiao X. Wang C. Zhang X. Li F. Zhou Y. (2012). Decreased microRNA-155 expression in ocular behcet’s disease but not in vogt koyanagi harada syndrome. Invest. Ophthalmol. Vis. Sci. 53 (9), 5665–5674. 10.1167/iovs.12-9832 22815348

[B266] Zhou R. Horai R. Mattapallil M. J. Caspi R. R. (2011). A new look at immune privilege of the eye: Dual role for the vision-related molecule retinoic acid. J. Immunol. 187 (8), 4170–4177. 10.4049/jimmunol.1101634 21918194 PMC3186879

[B267] Zhu S. Gong L. Li Y. Xu H. Gu Z. Zhao Y. (2019). Safety assessment of nanomaterials to eyes: An important but neglected issue. Adv. Sci. 6 (16), 1802289. 10.1002/advs.201802289 PMC6702629 31453052

[B268] Zhu Y. Liang H. Liu X. Wu J. Yang C. Wong T. M. (2021). Regulation of macrophage polarization through surface topography design to facilitate implant-to-bone osteointegration. Sci. Adv. 7 (14), eabf6654. 10.1126/sciadv.abf6654 33811079 PMC11060047

[B269] Zhuang Z. Zhang Y. Sun S. Li Q. Chen K. An C. (2020). Control of matrix stiffness using methacrylate-gelatin hydrogels for a macrophage-mediated inflammatory response. ACS Biomater. Sci. Eng. 6 (5), 3091–3102. 10.1021/acsbiomaterials.0c00295 33463297

[B270] Zimmer A. K. Maincent P. Thouvenot P. Kreuter J. (1994). Hydrocortisone delivery to healthy and inflamed eyes using a micellar polysorbate 80 solution or albumin nanoparticles. Int. J. Pharm. 110 (3), 211–222. 10.1016/0378-5173(94)90243-7

[B271] Zimmer A. Kreuter J. (1995). Microspheres and nanoparticles used in ocular delivery systems. Adv. Drug Deliv. Rev. 16 (1), 61–73. 10.1016/0169-409x(95)00017-2

[B272] Zirm E. (1906). Eine erfolgreiche totale Keratoplastik. Albr. Graefes Arch. für Ophthalmol. 64 (3), 580–593. 10.1007/bf01949227

[B273] Zolnik B. S. González-Fernández Á. Sadrieh N. Dobrovolskaia M. A. (2010). Minireview: Nanoparticles and the immune system. Endocrinology 151 (2), 458–465. 10.1210/en.2009-1082 20016026 PMC2817614

